# NEDD9, a novel target of miR-145, increases the invasiveness of glioblastoma

**DOI:** 10.18632/oncotarget.547

**Published:** 2012-08-05

**Authors:** Maria Carmela Speranza, Véronique Frattini, Federica Pisati, Dimos Kapetis, Paola Porrati, Marica Eoli, Serena Pellegatta, Gaetano Finocchiaro

**Affiliations:** ^1^ Unit of Molecular Neuro-Oncology, Fondazione I.R.C.C.S. Istituto Neurologico C. Besta, Milan, Italy; ^2^ Bioinformatics, Fondazione I.R.C.C.S. Istituto Neurologico C. Besta, Milan, Italy; ^3^ Dept Experimental Oncology, Campus IFOM-IEO, Milan, Italy

**Keywords:** miR-145, NEDD9, invasion, progression, glioma, glioblastoma

## Abstract

miR-145 is an important repressor of pluripotency in embryonic stem cells and a tumor suppressor in different cancers. Here, we found that miR-145 is strongly down-regulated in glioblastoma (GB) specimens and corresponding glioblastoma-neurospheres (GB-NS, containing GB stem-like cells) compared to normal brain (NB) and to low-grade gliomas (LGG). We observed a direct correlation between miR-145 expression and the progression-free survival (PFS) in LGG patients and overall survival (OS) in GB patients. Using microarray analysis, we identified relevant differences in gene expression profiles between GB-NS over-expressing miR-145 (miRover-NS) and GB-NS Empty (Empty-NS). We focused our attention on HEF1/Cas-L/NEDD9, a scaffold protein involved in invasion in several types of cancer. We confirmed a significant down-regulation of NEDD9 in miRover-NS and we found a higher expression in GB and GB-NS compared to NB. Approximately 50% of LGG patients expressed higher levels of NEDD9 than NB, and the PFS of such patients was shorter than in patients expressing lower levels of NEDD9. We observed that intracranial injection of GB-NS over-expressing miR-145 delays significantly tumor development: deriving tumors showed a significant down-regulation of NEDD9. In addition, we demonstrated a significant inhibition of invasion in silencing experiments with GB-NS shNEDD9 (shNEDD9), and an up-regulation of miR-145 in shNEDD9, suggesting a double-negative feedback loop between miR-145 and NEDD9. Our results demonstrate the critical role of miR-145 and NEDD9 in regulating glioblastoma invasion and suggest a potential role of NEDD9 as a biomarker for glioma progression.

## INTRODUCTION

Glioblastomas (GB) and other cancers may contain a populations of cells expressing stem cell programs and sharing expression patterns with embryonic stem cells [1]. Oct-4, Sox-2 and Nanog are core genes in embryonic stem cell maintenance and they are all up-regulated in GB and malignant gliomas [2]. Recent data suggest that their expression is tightly regulated by one microRNA, miR-145 [3].

MicroRNAs (miRNAs) are small non-coding RNA molecules with length of 20-22 nucleotides and are generated by the cleavage of 70-100 nucleotide hairpin pre-miRNA precursors [4]. miRNAs are able to regulate the expression of more than 30% of human genes via specific base pairing to the 3'-UTRs of messenger RNAs, which either blocks translation or promotes the degradation of the mRNA target. miRNAs are post-transcriptional modulators of gene expression and are involved in the regulation of several cellular processes, such as the cell cycle, apoptosis, proliferation and development. In particular, the abnormal expression of miRNAs is associated with several examples of human tumorigenesis: as such miRNAs may represent a novel, important class of oncogenes or tumor suppressor genes [5], [6]. MiR-145 is induced by the tumor suppressor gene TP53 through a p53 responsive element in its promoter and contributes to the silencing of the c-Myc oncogene [7], [8]. MiR-145 may also down-regulate the expression of MDM2, an E3 ubiquitin ligase promoting p53 degradation, creating a feedback loop with TP53 [9]. In mice, EGFR plays a negative role on miR-145 expression [10]. MiR-145 may also target VEGF-A expression in breast cancer [11].

This information and recent data indicate that miR-145 is a tumor suppressor capable of inhibiting proliferation in colon cancer and lung adenocarcinomas by targeting EGFR and NUDT1 [12–14]. Moreover, miR-145 is able to down-regulate several genes implicated in cell invasion, such as JAM-A, MUC1 and FSCN1, in breast, bladder and prostate cancer [15–18].

We have investigated the expression of miR-145 in gliomas. Our results show that its expression is down-regulated in these tumors and particularly in malignant subtypes, is associated to survival. We also identified a novel target of miR-145, NEDD9 and found that its regulation modulates the invasion potential of gliomas. Interestingly, NEDD9 and miR-145 expression appear reciprocally inter-connected.

## RESULTS

### miR-145 is strongly down-regulated in malignant gliomas

In previous unpublished studies, we attempted an in-depth characterization of the microRNA expression profiles of glioblastoma (GB) specimens, primary cell lines derived from GB growing as neurospheres (GB-NS) in the presence of b-fibroblast growth factor (b-FGF) and epidermal growth factor (EGF).

We evaluated the expression levels of different microRNAs previously known to be down-regulated in both tumors and stem cells: miR128a [15], let7a [16], miR181a [17], miR101 [18], and miR-145. miR128a is a typical brain-enriched miRNA that is usually up-regulated during differentiation and development; we found miR128a down-regulated in GB-NS compared to normal brain tissue. miRNA let7a is typically down-regulated in many cancers [19–21], whereas miR181 is mainly up-regulated during differentiation [22]. Both of these miRNAs are weakly expressed or down-regulated in GB [23]. miR101 is a well-known negative regulator of EZH2 [18], a histone methyltransferase of polycomb repressive complex 2 (PRC2) that catalyzes the trimethylation of histone H3 at lysine 27. H3K27 methylation causes gene silencing and is important for stem cell maintenance and proliferation [24], and miR101 was found to be moderately down-regulated in GB-NS. The most interesting result was found for miR-145; this miRNA, which is usually down-regulated in human embryonic stem cells and acts as a negative regulator of stemness [25–27], was expressed in low-grade gliomas but strongly down-regulated in both GB specimens and GB-NS.

**Figure 1:**
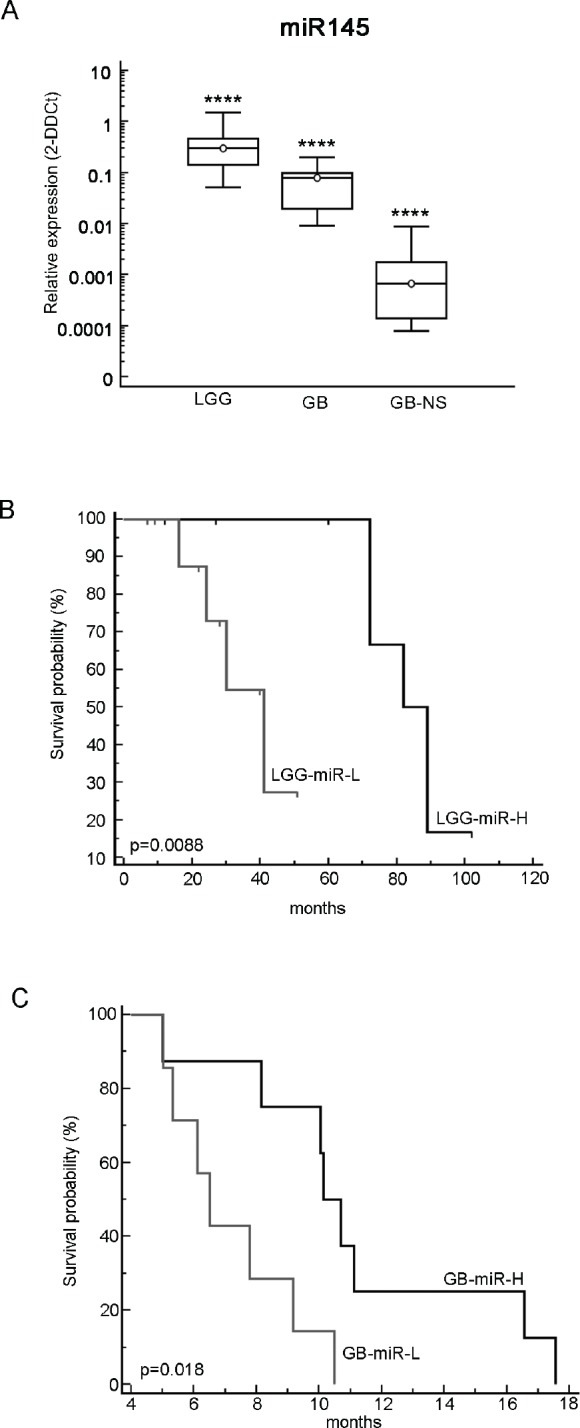
Characterization of miR-145 in malignant gliomas (A) A real-time PCR analysis was performed on 27 LGG samples, 18 GB samples and 18 corresponding GB-NS cell lines. The RNA data were normalized to the housekeeping gene RNU6B. miR-145 expression in LGG (mean ± SD: 0.41 ± 0.39, median: 0.3; P < 10^−9^) was significantly higher than in GB (mean ± SD: 0.07 ± 0.049, median: 0.08; P < 10^−10^) and in GB-NS (mean ± SD: 0.0019 ± 0.0029, median: 0.0007; P < 10^−10^) compared to normal brain samples (**** P < 0.0001). (B) Kaplan-Meier survival analysis showing the progression-free survival of 20 LGG patients with low miR-145 expression compared to other patients. (C) Kaplan-Meier survival analysis showing the OS of GB patients expressing high levels of miR-145 (n = 8) compared to GB with a lower expression of miR-145 (n = 7).

To investigate miR-145 expression in gliomas we studied 27 low-grade gliomas (LGG), 18 glioblastomas (GB) and 18 GB primary cell lines growing in culture as neurospheres (GB-NS). We found that miR-145 is expressed at much lower levels in GB compared to normal brain tissue and LGG (P < 10^−10^; p < 0.001, respectively). We also observed that miR-145 expression was very low or undetectable in GB-NS (P < 10^−10^ vs. normal brain; P < 10^−6^ vs. GB) (Figure 1A).

The expression of miR-145 was correlated with the progression-free survival (PFS) of LGG patients and with overall survival (OS) of GB patients. We compared LGG with higher and lower expression of miR-145 (LGG-miR-H, n = 10, relative expression vs. normal brain expressed as 2^−DDCt^ ≥ 0.3; LGG-miR-L, n = 10, 2^−DDCt^ < 0.3 vs. normal brain). LGG-miR-H patients showed significantly greater survival than LGG-miR-L (median PFS: 85.5 months in LGG-miR-H, 41 months in LGG-miR-L; P < 0.008) (Figure 1B). OS in LGG could not be evaluated, as the number of deceased patients was insufficient for the analysis. MiR-145 expression could be correlated with OS in a subgroup of 15 GB: GB with higher expression of miR-145 (GB-miR-H, 2^−DDCt^ ≥ 0.08, n = 8) had significantly longer survival than GB with lower expression of miR-145 (GB-miR-L, 2^−DDCt^ < 0.08, n = 7, P = 0.0183); median OS was 6.5 months in GB-miR-L and 10.4 months in GB-miR-H, (P = 0.018; Figure 1C). A trend for longer PFS in GB-miR-H was present (P < 0.09). These data indicate that low expression of miR-145 in gliomas is associated with a more aggressive phenotype.

### miR-145 affects glioblastoma stemness and migration

**Figure 2:**
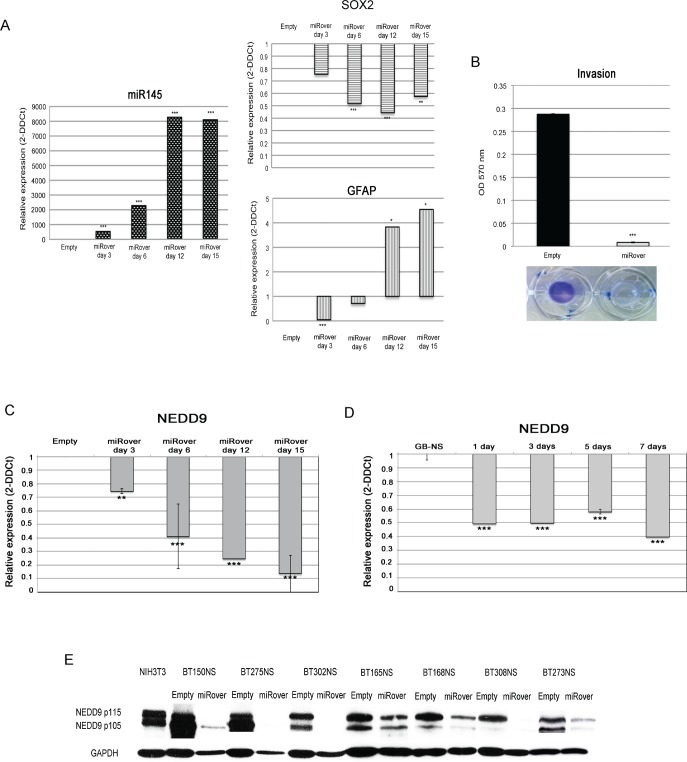
miR-145 in GB stem-like cells (A) Real-time PCR analysis showed, as expected, that miR-145 expression was significantly higher in miRover-NS (miRover) than in Empty-NS (Empty) (*** P < 0.001). A strong decrease in SOX2 (*** P < 0.001) and a concomitant increase of GFAP were found in miRover-NS vs. Empty-NS (* P < 0.05). (B) Significantly decreased invasion was observed in miRover-NS compared to Empty-NS in seven GB stem-like cell lines analyzed (*** P < 0.001). (C) Real-time PCR analysis demonstrated down-regulation of NEDD9 in miRover-NS 3- 15 days after infection and in GB-NS during differentiation taking place 1-7 days after addition of serum. The histogram is based on a representative cell line; the experiment was performed on a total of seven different miRover-NS, and NEDD9 showed the same behavior in all lines (** P < 0.01; *** P < 0.001). (D) Real-time PCR analysis demonstrated down-regulation of NEDD9 in GB-NS during differentiation taking place 1-7 days after addition of serum. The histogram represents the average of three different cell lines compared to normal brain. (E) Western blot analysis of NEDD9 expression in 7 GB-NS (BT150NS, BT275NS, BT302NS, BT165NS, BT168NS, BT308NS, and BT273NS) after infection with pCLNSX-miR-145 retroviral vector. Compared to Empty, the levels of NEDD9 protein are significantly reduced. GAPDH was used as housekeeping protein.

To verify the involvement of miR-145 in glioblastoma stemness and migration, we over-expressed miR-145 in seven GB-NS cell lines (Figure 2A, left panel), subsequently defined as miRover-NS. MiR-145 mRNA levels were up-regulated 3 days only after infection, and the level of expression significantly increased after 15 days.

First, we observed that in all GB-NS SOX2 was up-regulated (P = 0.0038), whereas the astrocyte marker GFAP was absent (P = 1.7×10^−10^). To assess the inverse relationship between expression of SOX2 and miR-145, we analyzed SOX2 levels in GB-NS following miR-145 over-expression. We found a significant decrease of SOX2 expression and a concomitant increase of GFAP in miRover-NS compared to cells infected with the empty vector (Empty-NS; P = 0.0001 and P = 0.04, respectively).

We also observed an increased level of miR-145 expression in GB-NS exposed to 3% serum. The expression pattern of SOX2 was symmetric, as it decreased after one day of exposure to serum (P = 0.005). Differentiation was also associated with an increased expression of GFAP (P = 0.0003) (Figure 2A, central panel).

Next, the effects of miR-145 over-expression on migration and proliferation were evaluated in miRover-NS and Empty-NS. MiRover-NS had a significantly lower migration capacity compared to Empty-NS (P = 6.4×10^−6^; Figure 2B).

An analysis of the proliferation kinetics revealed some decrease in cell proliferation in miRover-NS relative to Empty-NS but not significant (P = 0.6, data not shown) [28].

These data show that in miR-145 over-expression affects migration but not proliferation of GB-NS.

### miR-145 and NEDD9 expression are negatively correlated in GB-NS

To further characterize the effect of miR-145 on GB-NS, we compared gene expression profiles of miRover-NS and Empty-NS using the GeneChip Human Genome U133Plus Array, which includes over 47,000 human transcripts. We used a filter based on an expression value threshold of 100 and eliminated 32,438 of the 54,675 probe sets. The remaining 22,237 probe sets were used for the identification of DEGs using a fold change (FC) threshold. A total of 249 DEGs passed the FC cut-off in all the sample comparisons that were considered (Table S1). Functional classifications based on Gene Ontology annotations are listed in Table S2. We focused our validation experiments on NEDD9, as this gene is a putative target of miR-145 (mirSVR score of −0.766 on ww.microRNA.org). The potential for regulation of the 3' UTR of NEDD9 by miR-145 has been demonstrated in a bioinformatic study [29]. Moreover, the 3' UTR of NEDD9 exactly matches positions 2-8 of the mature miR-145 (8-mer, probability of conserved targeting – PCT > 0.75, www.TargetScanHuman.org).

Using real-time PCR, we observed that NEDD9 is down-regulated in miRover-NS compared to Empty-NS six days after infection (P = 0.0001) (Figure 2C). Down-regulation of NEDD9 expression was also demonstrated in GB-NS after serum exposure (P = 4.8×10^−5^) (Figure 2D).

The down-regulation of NEDD9 was also confirmed by Western blot analysis performed on seven samples of GB-NS over-expressing miR-145 relative to Empty-NS (Figure 2E).

Overall we found a strong inverse correlation between NEDD9 and miR-145 expression. These data support the assertion that miR-145 is responsible for NEDD9 regulation in GB-NS.

### miR-145 affects glioblastoma invasion by NEDD9 modulation in vivo

**Figure 3:**
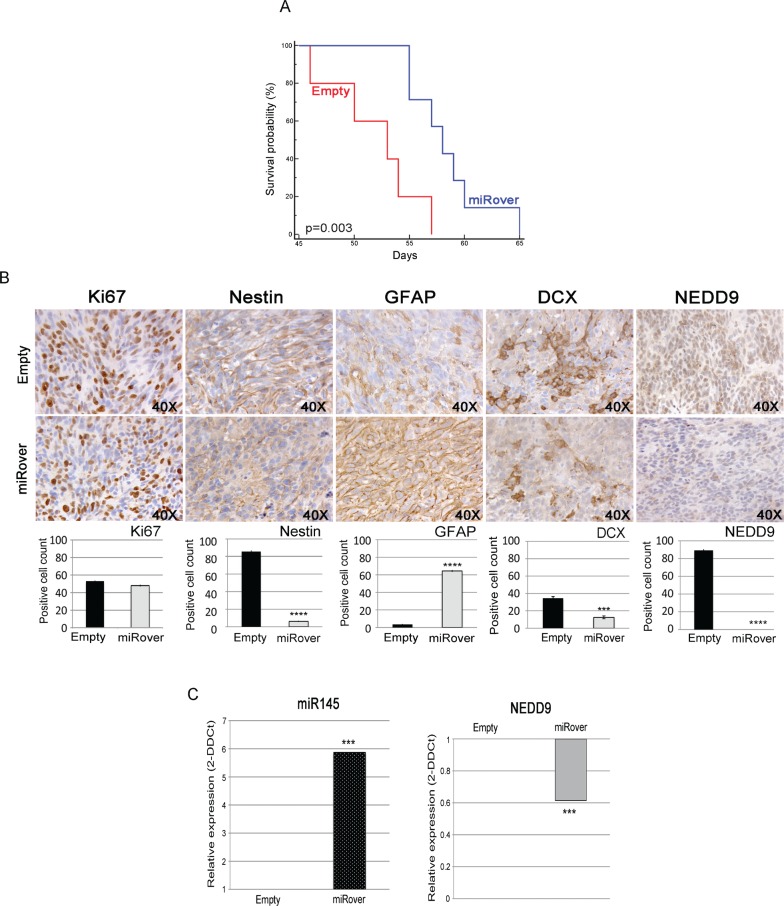
Effects of miR-145 overexpression in vivo (A) Kaplan-Meier survival analysis shows that mice injected with miRover-NS survive longer than mice injected with Empty-NS (P = 0.003). (B) Immunohistochemistry of miRover and Empty tumors (40X). Ki67-, Nestin-, GFAP, DCX- and NEDD9-positive cell were counted on three to five independent 40X fields per tumor in 2-3 animals per group. miRover tumors present a higher amount of GFAP-positive cells and a lower amount of cells positive for Ki67, Nestin, DCX and NEDD9 compared to Empty. Histograms represent the quantification of positive cells: Ki67 (48 ± 1.2% in miRover vs. 53.2 ± 0.6 in empty), Nestin (6.2 ± 0.7% in miRover vs. 85.7 ± 1.3 in empty), GFAP (64.4 ± 1.1% in miRover vs. 3.5 ± 0.5 in empty), DCX (12.5 ± 1.7% in miRover vs. 34.5 ± 1.9 in empty), and NEDD9 (0 ± 0% in miRover vs. 89.3 ± 1.2 in empty). A representative image for each tumor is displayed (* P < 0.05; *** P < 0.001; ****P < 0.0001). (C) Real-time PCR analysis of the expression of miR-145 and NEDD9 in tumors derived from miRover-NS relative to Empty-NS paraffin sections. The RNA inputs were normalized to the housekeeping gene RNU6B for miR-145 and beta-2 microglobulin for NEDD9. The over-expression of miR-145 and down-regulation of NEDD9 were maintained in vivo (*** P < 0.0001).

To elucidate the involvement of miR-145 in tumorigenesis, we stereotaxically implanted miRover-NS and Empty-NS into the nucleus caudatum of immunodeficient mice. The overexpression of miR-145 reduced the tumorigenicity of GB-NS, and mice injected with miRover-NS had a significantly higher survival rate (P = 0.003 vs. Empty-NS) (Figures 3A and S2). Histological and immunohistochemical analyses of the tumors derived from miRover-NS and Empty-NS showed a similar proliferation index measured by Ki67+ cells, in agreement with in vitro data. miRover tumors showed a significant down-regulation of the neural stem cell marker nestin, a strong up-regulation of GFAP and a significant decrease in the number of migrating cells evaluated as doublecortin (DCX) positive cells [30], [31] (P = 8.7×10^−8^, P = 4.9×10^−7^, and P = 0.0001, respectively, compared to Empty tumors). Notably, a significant reduction in tumor-invading cells, as measured in NEDD9+ cells, was detected in miRover tumors (P = 2.5×10^−8^ vs. Empty tumors) (Figure 3B). These data suggest that miR-145 overexpression reduces the invasion potential of GB-NS in vivo. A significantly higher expression of miR-145 was observed by real-time PCR performed on paraffin-embedded, serial sections derived from the miRover tumors (P = 1.59×10^−6^ vs. Empty tumors), indicating that overexpression of miR-145 was maintained during tumor formation. Concurrently, NEDD9 was significantly down-regulated (P = 10^−4^ vs. Empty tumors) (Figure 3C).

The over-expression of miR-145 affects the aggressive and invasive features of GB-NS, as confirmed by a greater differentiation of the tumor phenotype and a highly significant decrease in NEDD9, supporting the concept that miR-145 plays an important role in glioblastoma invasiveness by regulating NEDD9.

### NEDD9 expression affects low-grade glioma progression and glioblastoma invasiveness

**Figure 4:**
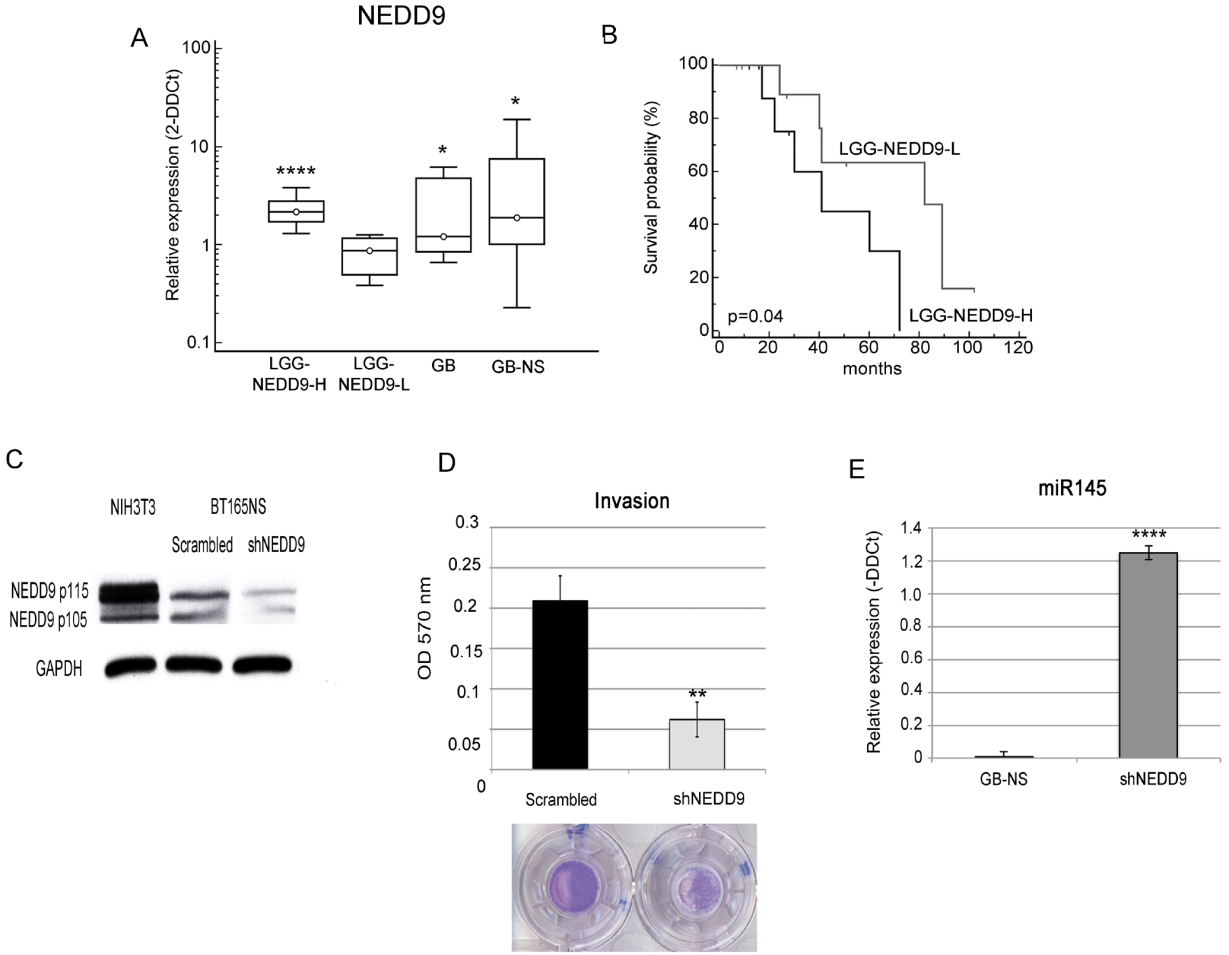
NEDD9 expression is associated to glioma survival and invasion (A) Real-time PCR analysis performed on 27 LGG and 18 GB and 18 GB-NS compared to normal brain: LGG-NEDD9-L, mean ± SD: 0.85 ± 0.33, p < 0.5; LGG-NEDD9-H, mean ± SD: 2.18 ± 0.85, P < 10^−4^; GB 2.55 ± 2.17 folds, P < 0.05; GB-NS mean ± SD: 4.45 ± 5.37, P 0.05. Data were normalized against the housekeeping gene beta-2 microglobulin. (* P < 0.05; **** P < 0.0001). (B) Kaplan-Meier survival analysis shows a significant correlation between the expression of NEDD9 and the PFS of LGG patients. LGG patients expressing lower levels of NEDD9 survived longer than LGG patients expressing higher levels of NEDD9 (median survival 41 months for LGG-NEDD9-L, 82 months for LGG-NEDD9-H, p<0.05). (C) Western blot analysis performed on a representative cell lines shows a significant reduction of NEDD9 expression in shNEDD9 cells compared to control cells (scrambled). GAPDH was used as a loading control. (D) The migration assay confirms a significant decrease in the migration ability in shNEDD9 relative to scrambled (** P < 0.01). (E) Real-time PCR on BT165NS-shNEDD9 (shNEDD9) shows an up-regulation of miR-145, which is expressed as in normal brain tissue, with a fold change > 1000 compared to BT165NS (GB-NS) (*** P < 0.001).

We studied the contribution of NEDD9 to glioma progression and invasiveness using real-time PCR to analyze mRNA expression levels in 18 GB specimens, 18 GB-NS cell lines and 27 LGG specimens previously investigated for expression of miR-145. NEDD9 was differentially expressed in GB (mean ± SD: 1.3 ± 2.5; P = 0.03 vs. normal brain) and in GB-NS (mean ± SD: 1.9 ± 4.5; P = 0.02 vs. normal brain) (Figure 4A).

LGG are characterized by the heterogeneous expression of NEDD9 (mean value 1.5 ± 0.8, median 1.3; P = 0.04 vs. normal brain). We distinguished two groups of LGG, identified by higher (LGG-NEDD9-H, 2^−DDCt^ ≥ 1.3, n = 11) and lower (LGG-NEDD9-L, 2^−DDCt^ < 1.3, n = 11) levels of NEDD9 expression (mean ± SD: 2.56 0.07 and 0.88 ± 0.04, respectively, P = 9.9×10^−6^).

We then compared the level of NEDD9 expression with the PFS in a subgroup of 22 LGG patients and found that LGG-NEDD9-H patients had a significantly lower survival than LGG-NEDD9-L patients (median OS: 41 months for LGG-NEDD9-L, 82 months for LGG-NEDD9-H; P < 0.05) (Figure 4B).

To confirm the role of NEDD9 in glioblastoma invasion, we performed silencing experiments using the same cell lines used for miR-145 over-expression. We observed a strong inhibition of NEDD9, as measured based on protein levels (Figure 4C), and real-time PCR (data not shown). We further evaluated in shNEDD9-NS and scrambled-NS the effect of NEDD9 inhibition on invasion. We observed a significant inhibition of invasion in shNEDD9-NS compared to scrambled-NS (P = 0.008) (Figure 4D). The proliferation kinetics based on three time points (48, 72 and 96 h) showed no significant differences between shNEDD9-NS and scrambled-NS (data not shown).

Notably, we found that shNEDD9-NS expressed miR-145 at levels comparable to that of normal brain and 1000-fold higher than in GB-NS (Figure 4E).

## DISCUSSION

MiR-145 is down-regulated in several types of cancers, such as bladder, urothelial, breast, prostate and colon carcinoma [32–35].

In our study, we found down-regulated expression of miR-145 in gliomas, confirming recent reports suggesting a tumor-suppressing role for this microRNA [27], [36]. We found that the expression levels of miR-145 are greatly reduced or absent in GB and in glioblastoma stem-like cells compared to normal brain, suggesting tumor suppressive functions for this microRNA. In particular, miR-145 is moderately down-regulated in low-grade gliomas but almost absent in GB and GB-NS. Interestingly, higher expression of this microRNA is correlated with better survival in LGG and GB patients, thereby confirming the involvement of miR-145 in glioma progression.

MiR-145 has been implicated in stemness maintenance. Fang et al [25] proposed a bistable system involving reciprocal interactions of SOX2 and miR-145 and suggested the involvement of miR-145 in GB stemness. Yang et al [27] demonstrated that miR-145 suppresses the self-renewal and tumor-initiating properties of GB stem-like cells both in vitro and in vivo. These studies support the concept that the miR-145-controlled pathways are important for reducing GB stem-like cells and their chemoradioresistance, partly via the downstream targets of miR-145 SOX2 and OCT4.

We confirmed this observation in primary cell lines derived from glioblastoma specimens growing in culture as neurospheres [37]. We found that the over-expression of miR-145 is correlated with a reduction in the expression of stem cell marker SOX2 and with increased expression of the astrocyte marker GFAP.

The over-expression of miR-145 in GB-NS also affects migration in vitro and in vivo, thus supporting a key role of miR-145 in GB invasion, as observed by Lee [36]. Because the invasive ability is one of the most important features of GB and one of the causes of poor prognosis, miR-145 appears to be an important factor for GB aggressiveness.

As a small, regulatory RNA, miR-145 has the potential to regulate several genes implicated in cell proliferation, apoptosis, stemness and invasion [6], [25], [26], [32], [33], [38], [39].

Gene expression profiling was used to identify three main signatures involved in stemness, apoptosis and invasion in GB-NS over-expressing miR-145.

We focused our validation experiments on the gene NEDD9 to test the relationship of miR-145 with invasion ability.

HEF1/CAS-L/NEDD9 is a non-catalytic scaffolding protein implicated in the invasion ability of several types of cancer [40–42]. NEDD9 has been proposed as a biomarker of invasiveness in lung cancer [43] and melanoma due to its role in the regulation and activation of transcriptional pathways relevant for metastasis and cancer progression, including FAK and Src [44]. The interactions of NEDD9 with FAK and Src lead to the tyrosine phosphorylation of NEDD9 to create binding sites for effector proteins such as Rac and the Cas-Crk complex, which have been previously studied in the context of cell migration [45]. In the past several years, NEDD9 has been studied in breast cancer, as a cancer cell-intrinsic protein with a pro-oncogenic role and as a candidate biomarker of tumor aggressiveness [40]. In GB, NEDD9 is a downstream effector of FAK that causes an increase in migration capacity [46]: in HNSCC, NEDD9 functions as an invasion regulator via VEGF activation [42].

We found that NEDD9 is up-regulated in malignant gliomas and in GB-NS, where the over-expression of miR-145 leads to the down-regulation of NEDD9.

NEDD9 is also expressed in a subgroup of low-grade glioma specimens and was show to be correlated with lower patient survival, indicating a relevant role of NEDD9 in glioma progression.

In vitro NEDD9 silencing in GB-NS is responsible for the significant inhibition of invasion ability, thereby confirming the involvement of NEDD9 in glioblastoma invasiveness. Finally, we demonstrated that NEDD9 silencing leads to the up-regulation of miR-145. Together, these results suggest that miR-145 down-regulates NEDD9, and NEDD9 down-regulates miR-145, forming a double-negative feedback loop in GB-NS. This hypothesis is supported by the presence of NEDD9-binding regions in the miR-145 locus, which would allow the direct binding of the NEDD9 3'UTR to the genomic region of miR-145 ([29] www.targetscan.org).

Recent studies [47] [48] support the idea that microRNAs can be regulated by their target interactions. This mechanism has led to the concept that “regulation in miRNA pathways is a two-way street”.

In our study, further investigations will be required to characterize the double-negative feedback loop between NEDD9 and miR-145.

The high levels of NEDD9 expression in GB and in LGG with lower PFS, along with the absence of NEDD9 in normal brain tissue, support the concept that NEDD9 expression is an important requisite for glioma invasion and progression.

## MATERIALS AND METHODS

### Tumor specimens and cell cultures

Primary glioblastomas (GB) and grade II gliomas, including fibrillary and gemistocytic astrocytomas (low-grade gliomas, LGG), were obtained from the Department of Neurosurgery of the “Istituto Neurologico Carlo Besta” with informed consent from the patients. Human glioma samples were frozen in liquid nitrogen and/or placed in saline solution immediately after surgery. GB cell lines (GB-NS) were obtained following the dissociation of GB specimens in collagenase type I (Invitrogen-Life Technologies, Carlsbad, California, USA) and were grown in DMEM/F12 (GIBCO- Life Technologies, Carlsbad, California/USA) with penicillin-streptomycin (1:100, EuroClone-Milan, Italy), B-27 supplement (1:50, GIBCO- Life Technologies, Carlsbad, California, USA), human recombinant fibroblast growth factor 2 (bFGF; 20 ng/mL; Tebu-bio, Milan, Italy), epidermal growth factor (EGF; 20 ng/mL; Tebu-bio, Milan, Italy) and heparin (5 microg/ml; Sigma Aldrich, St Louis, Missouri, USA).

### RNA extraction and reverse transcription

Total RNA was extracted from GB-NS, human frozen GB and LGG specimens using TRIzol® Reagent (Invitrogen) according to the manufacturer's instructions. Total RNA for miRNA analysis was reverse-transcribed using the TaqMan® microRNA Reverse Transcription Kit (Applied Biosystems-Life Technologies, Carlsbad, California, USA) with miRNA-specific primers. Total RNA for NEDD9 analysis was reverse-transcribed using the High Capacity cDNA Reverse Transcription Kit (Applied Biosystems-Life Technologies, Carlsbad, California, USA).

The extraction of total RNA from FFPE tissue sections was performed using the miRNeasy FFPE kit (Qiagen, Hilden, Germany) according to the manufacturer's instructions; the samples used for these extractions were obtained from the brains of mice injected with GB-NS over-expressing miR-145 (miRover-NS) or GB-NS Empty (Empty-NS).

### Real-time polymerase chain reaction analyses

Real-time PCR for the quantification of miR-145 was performed on an ABI PRISM 7900 real-time PCR system (Applied Biosystems, Foster City, CA, USA) with TaqMan chemistry (Applied Biosystems, Foster City, CA, USA) using 2.5 ng of cDNA from the RT-PCR solution in a final volume of 20 microL To quantify the mature miRNA and detect miR-145, we used the TaqMan® MicroRNA Assay kits (Applied Biosystems, Foster City, CA, USA) and a customized Assay on Demand (assay ID TM: 002278). We normalized miR-145 with respect to RNU6B (assay ID TM: 001093). The expression of NEDD9 was detected by SybrGreen chemistry (Forward: GAGCTGGATGGATGACTACGA; Reverse: AGCTCTTTCTGTTGCCTCTCA), and normalized relative to beta-2 microglobulin (Forward: GTGCTCGCGCTACTCTCTCT; Reverse: CCCAGACACATAGCAATTCAG). Commercial RNA from human normal brain (Ambion, AB) was used as the calibrator for the calculation of expression levels using the ΔΔCt method [49].

### Prediction of microRNA targets

To identify potential target genes and their conserved sites, we used the TargetScanHuman (release 5.2, http://www.targetscan.org), microRNA (www.microrna.org), MicroCosm Targets (www.ebi.ac.uk/enright.svr/microcosm/), PicTar (www.pictar.mdc-berlin.de), miRDB (www.mirdb.orf), and miRECORDS (www.mirecords.biolead.org) databases.

### Proliferation and Invasion Assay

Proliferation kinetics were measured by plating 8,000 cells/25 cm^2^. The cell count was obtained using the trypan blue exclusion test performed every 3 days (days 0, 3, 6, 9, and 12).

Invasion was assayed in vitro using the Transwell-96 system (Becton Dickinson, USA) as recommended by the manufacturer. After 24 h and 48 h, migrating cells were stained with crystal violet solubilized with 10% acetic acid, and the absorbance was determined at 570 nm.

### Western blot and antibodies

Protein samples were pelleted in RIPA lysis buffer with phosphatase and protease inhibitors, resolved using 10% SDS-PAGE and electroblotted onto nitrocellulose membranes. Membranes with transferred proteins were incubated with the primary antibody anti-NEDD9 (1:2000, Acris, Herford, Germany) or anti-GAPDH (1:5000, Abcam, Cambridge, UK). The primary antibody incubation was followed by incubation with the secondary antibody anti-mouse (1:10000). A chemiluminescence reaction using the ECL (enhanced chemiluminescence) Plus kit (Amersham, GE Healthcare, Buckinghamshire, UK) was detected using film.

### Plasmids and Infection

pCLNSX-miR-145 and pCLNSX-Empty were used to transfect packaging cells using Phoenix Ampho. After 48 h of transfection, we infected GB-NS with supernatant containing virions with miR-145 cDNA or the empty vector (miRover-NS or empty-NS, respectively). The infection was repeated twice, and cells were selected for resistance to Neomycin (0.4 mg/ml).

shNEDD9 plasmid DNA (Mission RNAi, TRCN000004967, Sigma-Aldrich, St Louis, Missouri, USA) for NEDD9 silencing was used for transient transfection according to the manufacturer's instructions. The transfection was performed using 5 microgrammi of shNEED9/Scrambled plasmids for every 200000 primary GB-NS cells. The cell pellets for RNA and protein analyses were collected at 48 h after transfection.

### Microarray analysis

Microarray analysis was performed on five different NS cell lines overexpressing miR-145, and Empty-NS cells were used as controls.

Fragmented cRNAs were hybridized to the HG-U133 Plus 2.0 GeneChip (Affymetrix, Santa Clara, USA) following standard procedures. Data processing was mainly performed using Bioconductor 2.10 and R.15 [50]. The Robust Multichip Average [51] algorithm was applied to normalize using the quantile method, and normalized probeset intensities were calculated. A signal-based filtering was applied to the expression level (>100) of each probeset for all of the different groups that were considered. Differentially expressed genes were identified using a fold change (FC) threshold of 1.2 for all sample comparisons. The functional annotation of genes that passed the FC and expression signal cut-offs was performed using the Gene Ontology (GO) Biological Process category and the hypergeometric test (hyperGTest function) [50] for gene over-representation.

### In vivo experiments

A total of 45 immune-deficient CD1-nude mice received brain injections of 10^5^ miRover-NS or Empty-NS cells (n = 10/group for survival, n = 5/group for histological studies) from four GB-NS cell lines (BT165NS, BT168NS, BT273NS, BT275NS) infected with retroviral vectors (pCLNSX-miR-145 and pCLNSX-Empty).

### Immunohistochemistry and immunofluorescence analyses

Immunohistochemical analyses for Ki67 (1:100, BD, New Jersey, USA), Nestin (1:100, R&D System, Minnesota, USA), GFAP (1:100, DAKO, Glostrup, Denmark), DXC (Abcam, 1:100) and NEDD9 (Acris, 1:50) were performed on paraffin-embedded sections.

Tumor sections were blocked with 5% goat serum in PBS for 60 min, incubated overnight with primary antibodies, and incubated with biotinylated secondary antibodies (1:200 Vector Lab) for 1 h. Antibody binding was detected using the Vectastain Elite Avidin–Biotin Complex-Peroxidase kit according to the manufacturer's instructions.

All sections were counterstained with Mayer's hematoxylin and visualized using a LEICA MDLB light microscope.

The percentages of Ki67-, GFAP-, and NEDD9-positive cells were calculated in 5 independent high-magnification fields. Positive cells were counted only within the tumor area. The results are expressed as percentages.

The positive rates were counted manually in triplicate by two observers (F.P. and MC.S.) using the photomicrographs.

### Statistical analysis

Statistical comparisons of the data sets were performed using a two-tailed Student's T-test, and the results were considered significant at p<0.05.

Cumulative survival curves were constructed using the Kaplan-Meier method (MedCalc 9.3).

## Supplementary Tables and Figures




