# Supplementary material for: NEDD9, a novel target of miR-145, increases the invasiveness of glioblastoma
Source: Oncotarget. 2012 Aug 5;3(7):723–34. doi: 10.18632/oncotarget.547 (PMC3443255; doi:10.18632/oncotarget.547)
Supplement: Supplementary file 1 [file oncotarget-03-723-s001.docx]

**NEDD9, a novel target of miR-145, increases the inasiveness of glioblastoma-Speranza et al**

| **Ranking** | **Probeset ID** | **miRover-NS1** | **Empty-NS1** | **miRover-NS2** | **Empty-NS2** | **miRover-NS3** |
| --- | --- | --- | --- | --- | --- | --- |

**Supplementary Table1:**

| 1 | 1552275_s_at | 59.93 | 41.22 | 65.98 | 51.39 | 48.22 |
| --- | --- | --- | --- | --- | --- | --- |
| 2 | 1552507_at | 103.77 | 80.08 | 97.88 | 38.21 | 26.74 |
| 3 | 1552767_a_at | 183.81 | 11.14 | 377.96 | 1030.97 | 1206.6 |
| 4 | 1555225_at | 285.36 | 233.31 | 215.75 | 151.98 | 152.69 |
| 5 | 1555778_a_at | 36.07 | 5.45 | 100.85 | 12.2 | 15.03 |
| 6 | 1556037_s_at | 25.65 | 15.17 | 19.45 | 45.08 | 29.04 |
| 7 | 1556051_a_at | 122.13 | 95.02 | 201.3 | 344.39 | 237.05 |
| 8 | 1556203_a_at | 103.24 | 80.67 | 75.28 | 157.38 | 108.24 |
| 9 | 1558387_at | 5.92 | 4.49 | 8.25 | 46.41 | 7.67 |
| 10 | 1558692_at | 189.19 | 266.96 | 175.28 | 30.2 | 224.3 |
| 11 | 1560562_a_at | 72.52 | 88.67 | 109.28 | 167.28 | 197.46 |
| 12 | 1564233_at | 33.54 | 25.27 | 60.6 | 281.25 | 42.29 |
| 13 | 1564494_s_at | 80.06 | 46.36 | 106.26 | 307.94 | 103.69 |
| 14 | 201124_at | 101.89 | 29.55 | 136.67 | 235.8 | 240.56 |
| 15 | 201243_s_at | 766.94 | 940.1 | 771.77 | 1686.15 | 702.73 |
| 16 | 201325_s_at | 270.51 | 22.85 | 629.34 | 1877.08 | 678.2 |
| 17 | 201452_at | 108.22 | 88.34 | 94.15 | 60.62 | 64.88 |
| 18 | 201471_s_at | 821.13 | 1018.56 | 839.47 | 510.04 | 843.75 |
| 19 | 201564_s_at | 327.59 | 666.08 | 295.64 | 625.17 | 378.58 |
| 20 | 201694_s_at | 731.57 | 216.46 | 1361.9 | 2758.3 | 2519.42 |
| 21 | 201842_s_at | 33.05 | 50.01 | 19 | 14.76 | 320.97 |
| 22 | 201939_at | 327.89 | 616.2 | 208.35 | 14.2 | 101.37 |
| 23 | 201983_s_at | 262.81 | 132.13 | 464.77 | 1074.83 | 547.11 |
| 24 | 202149_at | 327.58 | 393.54 | 476.08 | 1145.3 | 315.89 |
| 25 | 202272_s_at | 505.38 | 757 | 452.18 | 376.19 | 432.07 |
| 26 | 202330_s_at | 888.07 | 1417.26 | 798.1 | 1105.51 | 957.08 |
| 27 | 202628_s_at | 43.61 | 30.43 | 67.64 | 297.08 | 79.31 |
| 28 | 202672_s_at | 165.6 | 204.64 | 142.53 | 53.36 | 83.72 |
| 29 | 202847_at | 74.72 | 111.8 | 81.73 | 52.68 | 34.69 |
| 30 | 202897_at | 257.03 | 386.47 | 252.67 | 176.38 | 161.55 |
| 31 | 203096_s_at | 114.59 | 144.56 | 98.7 | 50.7 | 53.07 |
| 32 | 203123_s_at | 292.1 | 359.6 | 305.17 | 180.68 | 283.63 |
| 33 | 203124_s_at | 438.53 | 631.18 | 449.55 | 277.47 | 404.48 |
| 34 | 203304_at | 310.38 | 32.05 | 802.75 | 2675.11 | 326.45 |
| 35 | 203350_at | 135.57 | 238.21 | 121.32 | 163.98 | 239.22 |
| 36 | 203408_s_at | 43.14 | 13.06 | 93.37 | 63.64 | 17.58 |
| 37 | 203413_at | 105.99 | 78.14 | 156.26 | 66.31 | 56.7 |

| 42 | 203637_s_at | 222.03 | 318.47 | 221.29 | 339.15 | 231.69 |
| --- | --- | --- | --- | --- | --- | --- |
| 43 | 203665_at | 191.29 | 356.94 | 141.45 | 61.31 | 167.07 |
| 44 | 203786_s_at | 101.34 | 144.68 | 82.38 | 23.46 | 60.51 |
| 45 | 203870_at | 215.28 | 261.34 | 277.66 | 776.88 | 336.65 |
| 46 | 203925_at | 821.79 | 1148.2 | 786.7 | 535.54 | 802.04 |
| 47 | 204075_s_at | 121.37 | 160.57 | 123.65 | 154.27 | 135.27 |
| 48 | 204135_at | 159.68 | 309.98 | 108.5 | 145.82 | 68.85 |
| 49 | 204398_s_at | 95.35 | 75.64 | 94.61 | 148.68 | 130.68 |
| 50 | 204435_at | 197.94 | 246.53 | 233.68 | 341.95 | 308.57 |
| 51 | 204526_s_at | 341.1 | 280.65 | 442.8 | 259.81 | 163.19 |
| 52 | 204595_s_at | 4157.37 | 5575.58 | 3827.13 | 115.31 | 83.72 |
| 53 | 204597_x_at | 1186.21 | 1505.7 | 997.28 | 27.94 | 14.31 |
| 54 | 204627_s_at | 27.76 | 17.81 | 37.63 | 103.83 | 19.91 |
| 55 | 204851_s_at | 283.08 | 109.08 | 414.86 | 665.37 | 275.94 |
| 56 | 204932_at | 31.29 | 18.53 | 30.91 | 39.43 | 22.33 |
| 57 | 205129_at | 260.93 | 176.22 | 173.18 | 92.91 | 144.73 |
| 58 | 205143_at | 382.07 | 246.9 | 377.95 | 166.59 | 58.28 |
| 59 | 205249_at | 198.09 | 78.78 | 309.7 | 389.21 | 138.46 |
| 60 | 205363_at | 43.08 | 9.03 | 95.65 | 135.55 | 244.85 |
| 61 | 205372_at | 37.34 | 45.06 | 38.38 | 14.32 | 12.18 |
| 62 | 205479_s_at | 716.96 | 1146.03 | 599.24 | 152.32 | 34.16 |
| 63 | 205991_s_at | 33.97 | 23.22 | 36.62 | 73.02 | 64.43 |
| 64 | 206071_s_at | 140.33 | 98.87 | 189.28 | 246.34 | 91.51 |
| 65 | 206115_at | 178.71 | 94.72 | 383.59 | 302.68 | 209.21 |
| 66 | 206172_at | 460.53 | 131.66 | 1005.92 | 1942.18 | 11.57 |
| 67 | 206695_x_at | 402.46 | 533.08 | 401.69 | 321.19 | 488.47 |
| 68 | 207002_s_at | 8.77 | 6.45 | 11.7 | 48.13 | 56.13 |
| 69 | 207012_at | 104.05 | 25.05 | 150.11 | 357.78 | 112.09 |
| 70 | 207327_at | 114.94 | 184.02 | 142.96 | 266.39 | 303.31 |
| 71 | 207604_s_at | 38.97 | 117.48 | 48.83 | 67.07 | 59.32 |
| 72 | 207983_s_at | 245.89 | 107.09 | 314.59 | 725.86 | 884.62 |
| 73 | 208325_s_at | 111.08 | 54.15 | 126.67 | 198.28 | 94.4 |
| 74 | 209119_x_at | 90.6 | 148.2 | 79.71 | 34.91 | 44.19 |
| 75 | 209283_at | 554.8 | 700.48 | 858.12 | 103.58 | 547.95 |
| 76 | 209306_s_at | 233.56 | 414.97 | 231.23 | 516.69 | 426.62 |
| 77 | 209307_at | 395.17 | 653.18 | 397.58 | 772.52 | 768.66 |
| 78 | 209383_at | 467.48 | 578.88 | 526.13 | 172.6 | 448.44 |
| 79 | 209684_at | 345.92 | 529.19 | 287.99 | 94.05 | 318.58 |
| 80 | 209750_at | 233 | 190.32 | 291.29 | 401.73 | 112.68 |
| 81 | 209840_s_at | 38.03 | 10.59 | 76.9 | 543.04 | 1501.38 |
| 82 | 209841_s_at | 41.14 | 11.67 | 82.5 | 629.08 | 1656.98 |
| 83 | 209921_at | 309.97 | 454.53 | 316.18 | 97.83 | 177.43 |
| 84 | 209955_s_at | 121.79 | 72.38 | 161.02 | 29.79 | 29.85 |

| 85 | 210018_x_at | 713.39 | 565.84 | 932.21 | 372.28 | 132.27 |
| --- | --- | --- | --- | --- | --- | --- |
| 86 | 210090_at | 178.78 | 289.63 | 139.26 | 168.72 | 260.25 |
| 87 | 210095_s_at | 527.88 | 73 | 1141.52 | 2721.51 | 641.48 |
| 88 | 210138_at | 340.24 | 277.27 | 314.42 | 11.95 | 221.28 |
| 89 | 210407_at | 96.08 | 79.16 | 111.28 | 142.88 | 92.32 |
| 90 | 210882_s_at | 52.88 | 43.73 | 56.71 | 98.74 | 130.77 |
| 91 | 210933_s_at | 54.44 | 91.18 | 52.13 | 104.11 | 61.41 |
| 92 | 211748_x_at | 336.35 | 503.54 | 256.88 | 83.85 | 74.35 |
| 93 | 212067_s_at | 53.33 | 65.15 | 69.12 | 142.28 | 154.07 |
| 94 | 212365_at | 524.29 | 650.64 | 350.11 | 62.03 | 36.85 |
| 95 | 212614_at | 143.42 | 180.62 | 207.11 | 127.32 | 437.83 |
| 96 | 212812_at | 2531.72 | 3288.64 | 2590.48 | 4689.83 | 1430.23 |
| 97 | 213050_at | 550.92 | 734.96 | 519.14 | 385.79 | 810.7 |
| 98 | 213355_at | 93.36 | 58.28 | 168.68 | 282.38 | 522.78 |
| 99 | 213436_at | 57.9 | 72.45 | 73.79 | 182.35 | 372.73 |
| 100 | 213438_at | 179.09 | 219.36 | 122.59 | 28.27 | 82.86 |
| 101 | 213533_at | 64.88 | 86.52 | 61.1 | 148.4 | 77.57 |
| 102 | 213601_at | 418.68 | 145.63 | 844.79 | 348.13 | 336.43 |
| 103 | 213763_at | 218.03 | 157.03 | 266.58 | 204.42 | 287.6 |
| 104 | 213830_at | 541.13 | 288.63 | 577.94 | 227.56 | 118.75 |
| 105 | 213872_at | 1525.46 | 1074.87 | 1436.27 | 1165.82 | 545.33 |
| 106 | 214436_at | 27.76 | 22.63 | 24.34 | 70.51 | 134.54 |
| 107 | 214841_at | 202.59 | 137.33 | 405.52 | 815.04 | 258.38 |
| 108 | 214844_s_at | 72.58 | 107.18 | 98.89 | 389.32 | 272 |
| 109 | 214913_at | 674.03 | 498.19 | 1174.58 | 3990.56 | 488.56 |
| 110 | 215025_at | 30.91 | 19.48 | 49.02 | 120.29 | 106.47 |
| 111 | 216379_x_at | 266.51 | 337.44 | 234.89 | 180.07 | 198.74 |
| 112 | 216867_s_at | 113.17 | 65.96 | 218.81 | 358 | 706.31 |
| 113 | 217168_s_at | 990.43 | 1275.71 | 898.76 | 300.9 | 1231.08 |
| 114 | 217540_at | 111.51 | 199.46 | 106.94 | 75.58 | 70.82 |
| 115 | 217678_at | 240.39 | 319.39 | 253.05 | 80.4 | 117.47 |
| 116 | 217739_s_at | 1097.19 | 1332.06 | 1080.39 | 215.46 | 505.58 |
| 117 | 217757_at | 667.49 | 103.33 | 1483.13 | 2171.88 | 306.08 |
| 118 | 217887_s_at | 641.25 | 782.48 | 699.81 | 562.64 | 894.56 |
| 119 | 217901_at | 144.58 | 8.72 | 256.88 | 580.67 | 127.49 |
| 120 | 218006_s_at | 191.87 | 144.69 | 170.94 | 216.85 | 370.27 |
| 121 | 218065_s_at | 399.89 | 495 | 487.12 | 1182.36 | 633.41 |
| 122 | 218748_s_at | 110.8 | 88.62 | 76.42 | 111.63 | 79.06 |
| 123 | 218972_at | 150.99 | 185.92 | 200.13 | 164.08 | 129.73 |
| 124 | 219049_at | 107.36 | 17.17 | 220.98 | 564.43 | 294.17 |
| 125 | 219073_s_at | 61.28 | 34.96 | 97.32 | 372.82 | 55.48 |

| 131 | 220342_x_at | 176.75 | 140.46 | 224.23 | 164.09 | 153.45 |
| --- | --- | --- | --- | --- | --- | --- |
| 132 | 220543_at | 48.02 | 20.75 | 100.18 | 380.73 | 166.75 |
| 133 | 221558_s_at | 334.88 | 19.33 | 629.59 | 1368.77 | 449.03 |
| 134 | 221805_at | 722.93 | 1033 | 1028.03 | 7.91 | 6.35 |
| 135 | 222108_at | 17.24 | 9.68 | 25.17 | 59.56 | 322.21 |
| 136 | 222293_at | 72.72 | 43.63 | 106.59 | 167.45 | 103.76 |
| 137 | 222525_s_at | 521.85 | 821.56 | 531.6 | 667.5 | 215.13 |
| 138 | 222787_s_at | 597.06 | 346.24 | 579.69 | 895.01 | 1265.85 |
| 139 | 223044_at | 68.35 | 8.17 | 143.22 | 295.94 | 220.11 |
| 140 | 223923_at | 66.15 | 49.59 | 126.49 | 362.68 | 93.6 |
| 141 | 224250_s_at | 320.98 | 390.68 | 332.25 | 781.46 | 531.64 |
| 142 | 224480_s_at | 10.28 | 13.6 | 14.59 | 18.05 | 8.5 |
| 143 | 224559_at | 277.06 | 907.8 | 358.58 | 281.17 | 326.04 |
| 144 | 224646_x_at | 661.48 | 25.31 | 1256.41 | 684.98 | 116.52 |
| 145 | 224802_at | 660.91 | 849.64 | 629.98 | 1326.08 | 523.82 |
| 146 | 224943_at | 84.37 | 106.94 | 108.68 | 134.35 | 111.9 |
| 147 | 224985_at | 1137.17 | 1377.66 | 1057.9 | 1417.58 | 1285.24 |
| 148 | 224997_x_at | 141.87 | 22.14 | 242.87 | 147.72 | 26.24 |
| 149 | 225150_s_at | 68.94 | 122.08 | 64.48 | 181.27 | 140.46 |
| 150 | 225299_at | 79.51 | 27.68 | 144.05 | 255.87 | 18.68 |
| 151 | 225301_s_at | 225.06 | 139.44 | 360.8 | 461.18 | 87.98 |
| 152 | 225351_at | 586.72 | 947.87 | 579.37 | 288.9 | 483.37 |
| 153 | 225436_at | 749.7 | 1498.17 | 721.16 | 1454.08 | 585.02 |
| 154 | 225566_at | 402.86 | 287.11 | 545.66 | 737.97 | 213.35 |
| 155 | 225599_s_at | 77.87 | 114.01 | 50.88 | 41.27 | 51.88 |
| 156 | 225603_s_at | 144.62 | 209.61 | 135.24 | 88.66 | 143.08 |
| 157 | 225664_at | 66.11 | 11.67 | 209.93 | 149.63 | 12.62 |
| 158 | 225814_at | 639.97 | 848.57 | 642.08 | 447.29 | 412.88 |
| 159 | 226186_at | 145.29 | 256.97 | 134.58 | 395.67 | 220.63 |
| 160 | 226415_at | 286.66 | 182.51 | 226.14 | 19.48 | 38.42 |
| 161 | 226545_at | 333.57 | 53.77 | 890.14 | 1985.29 | 87.45 |
| 162 | 227148_at | 208.65 | 124.32 | 318.68 | 793.5 | 519.98 |
| 163 | 227152_at | 130.84 | 99.35 | 117.78 | 75.65 | 75.16 |
| 164 | 227367_at | 74.38 | 102.25 | 79.34 | 49.61 | 81.3 |
| 165 | 227478_at | 201.37 | 124.9 | 315.56 | 567.66 | 318.62 |
| 166 | 227620_at | 264.34 | 98.48 | 467.49 | 963.07 | 704.33 |
| 167 | 227627_at | 25.21 | 19.57 | 26.75 | 103.62 | 88.41 |
| 168 | 227760_at | 353.45 | 470.86 | 306.7 | 14.76 | 41.33 |
| 169 | 228188_at | 67.11 | 41.57 | 108.18 | 159.26 | 37.13 |
| 170 | 228256_s_at | 16.38 | 11.24 | 12.51 | 32.03 | 87.76 |

| 175 | 229004_at | 19.45 | 12.84 | 36.17 | 169.88 | 28.77 |
| --- | --- | --- | --- | --- | --- | --- |
| 176 | 229225_at | 266.36 | 120.92 | 338.48 | 443.11 | 101.44 |
| 177 | 229272_at | 114.36 | 60.42 | 99.01 | 80.07 | 48.88 |
| 178 | 229376_at | 488.71 | 614.33 | 552.38 | 1025.72 | 643.58 |
| 179 | 229801_at | 195.42 | 130.83 | 162.57 | 77.7 | 68.68 |
| 180 | 230030_at | 152.07 | 8.44 | 299.39 | 932.29 | 963.9 |
| 181 | 230144_at | 101.78 | 5.5 | 206.65 | 407.99 | 103.14 |
| 182 | 230147_at | 16.51 | 28.04 | 16.27 | 10.85 | 13.05 |
| 183 | 230425_at | 212.02 | 318.68 | 203.76 | 706.68 | 198.78 |
| 184 | 231513_at | 124.02 | 83.86 | 121.48 | 236.08 | 34.56 |
| 185 | 231576_at | 202.18 | 125.58 | 196.35 | 133.27 | 74.55 |
| 186 | 231697_s_at | 36.74 | 28.85 | 83.69 | 103.53 | 27.91 |
| 187 | 231729_s_at | 46.56 | 36.41 | 57.69 | 212.39 | 55.05 |
| 188 | 231735_s_at | 217.96 | 361.88 | 268.27 | 352.18 | 332.2 |
| 189 | 232231_at | 40.49 | 25.69 | 63.54 | 11.1 | 15.26 |
| 190 | 232270_at | 44.08 | 33.28 | 38.83 | 201.56 | 41.55 |
| 191 | 234314_at | 188.27 | 147.96 | 148.7 | 6.98 | 8.45 |
| 192 | 234986_at | 486.02 | 695.94 | 410.55 | 220.32 | 419.32 |
| 193 | 235049_at | 158.05 | 130.86 | 158.23 | 115.34 | 176.67 |
| 194 | 235213_at | 269.12 | 397.38 | 298.4 | 141.47 | 183.82 |
| 195 | 235311_at | 190.66 | 146.7 | 167.33 | 106.11 | 148.12 |
| 196 | 235551_at | 106.46 | 129.68 | 115.48 | 41.24 | 54.05 |
| 197 | 236047_at | 43.88 | 55.81 | 27 | 12.9 | 42.65 |
| 198 | 236133_x_at | 81.7 | 123.48 | 87.17 | 55.84 | 55.43 |
| 199 | 236140_at | 538.61 | 651.68 | 381.86 | 202.69 | 402.01 |
| 200 | 236291_at | 37.9 | 46.54 | 41.14 | 67.62 | 162.79 |
| 201 | 236344_at | 65.36 | 18.1 | 131.23 | 317.51 | 58.84 |
| 202 | 237833_s_at | 29.27 | 19.1 | 27.85 | 35.86 | 96.19 |
| 203 | 238739_at | 67.12 | 132.44 | 65.06 | 48.25 | 33.89 |
| 204 | 238898_at | 181.62 | 151.25 | 160.85 | 57.69 | 47.69 |
| 205 | 241741_at | 113.88 | 93.57 | 119.91 | 178.27 | 63.26 |
| 206 | 241922_at | 110.25 | 78.47 | 106.4 | 274.3 | 70.75 |
| 207 | 242488_at | 176.98 | 12.23 | 496.45 | 1603.43 | 205.21 |
| 208 | 242825_at | 51.87 | 16.39 | 85.67 | 454.81 | 235.94 |
| 209 | 243495_s_at | 242.68 | 129.7 | 260.88 | 186.64 | 107.28 |
| 210 | 243606_at | 141.58 | 216.22 | 135.42 | 87.73 | 107.59 |
| 211 | 244043_at | 103.16 | 263.13 | 81.73 | 53.49 | 195.19 |
| 212 | 244163_at | 18.98 | 6.19 | 35.96 | 132.88 | 37.13 |
| 213 | 244227_at | 43.7 | 30.44 | 53.15 | 251.29 | 47.36 |
| 214 | 244849_at | 19.73 | 10.85 | 26.84 | 161.08 | 24.06 |

| **Empty-NS3** | **miRover-NS4** | **Empty-NS4** | **miRover-NS5** | **Empty-NS5** | **FC_NS1** |
| --- | --- | --- | --- | --- | --- |

| 59.69 | 52.04 | 42.54 | 81.52 | 108.88 | -1.449275362 |
| --- | --- | --- | --- | --- | --- |
| 41.6 | 45.42 | 59.29 | 73.58 | 136.44 | -1.298701299 |
| 951.53 | 158 | 120.58 | 1417.91 | 605.98 | -16.66666667 |
| 108.07 | 525.68 | 436.55 | 109.88 | 89.28 | -1.219512195 |
| 24.25 | 4.78 | 7.16 | 1366.19 | 2073.73 | -6.666666667 |
| 15.3 | 132.17 | 87.25 | 31.57 | 25.17 | -1.694915254 |
| 337.08 | 87.47 | 124.9 | 289.31 | 387.27 | -1.282051282 |
| 137.86 | 124.52 | 97.42 | 126.78 | 179.61 | -1.282051282 |
| 9.95 | 5.04 | 4.17 | 146.74 | 251.28 | -1.315789474 |
| 297.42 | 284.52 | 345.12 | 128.6 | 197.82 | 1.41 |
| 252.25 | 68.57 | 93.88 | 205.18 | 262.87 | 1.22 |
| 27.5 | 20.85 | 27.48 | 34.18 | 27.82 | -1.333333333 |
| 155.18 | 115.35 | 148.01 | 126.34 | 152.3 | -1.724137931 |
| 161.59 | 45.36 | 31.77 | 64.58 | 34.6 | -3.448275862 |
| 545.61 | 601 | 794.19 | 740.42 | 918.64 | 1.23 |
| 539.92 | 10.87 | 13.12 | 2425.77 | 1850.13 | -12.5 |
| 52.69 | 94.47 | 75.23 | 38.36 | 46.46 | -1.219512195 |
| 1079.41 | 1240.31 | 1700.08 | 1172.6 | 1507.05 | 1.24 |
| 607.91 | 361.38 | 670.24 | 466.1 | 858.36 | 2.03 |
| 2079.53 | 218.62 | 167.28 | 1110.57 | 574 | -3.333333333 |
| 674.08 | 24.37 | 45.48 | 48.27 | 90.71 | 1.51 |
| 154.24 | 466.22 | 646.97 | 331.72 | 662.43 | 1.88 |
| 693.92 | 126.92 | 104.47 | 184.93 | 153.2 | -2 |
| 537.46 | 326.04 | 432.07 | 1899.09 | 2611.58 | 1.2 |
| 602.93 | 669.67 | 826.58 | 445.78 | 548.68 | 1.5 |
| 1264.97 | 1098 | 1571.68 | 453.92 | 696.68 | 1.6 |
| 96.01 | 44.22 | 29.81 | 113.09 | 240.31 | -1.428571429 |
| 128.15 | 71.46 | 114.05 | 211.04 | 339.71 | 1.24 |
| 58.52 | 68.68 | 89.17 | 37.38 | 30.47 | 1.5 |
| 249.93 | 163.57 | 248.18 | 288.69 | 507.85 | 1.5 |
| 71.96 | 115.58 | 140.37 | 96.25 | 154.3 | 1.26 |
| 379.13 | 215.36 | 280.71 | 152.06 | 378.15 | 1.23 |
| 569.76 | 361.71 | 513.51 | 334.22 | 661.75 | 1.44 |
| 253.36 | 65.78 | 53.34 | 232.52 | 163.3 | -10 |
| 370.26 | 140.35 | 220.29 | 314.36 | 453.52 | 1.76 |
| 14.31 | 10.17 | 12.54 | 219.18 | 272.81 | -3.333333333 |
| 81.31 | 439.34 | 610.4 | 116.08 | 153.12 | -1.351351351 |
| 808.67 | 736.78 | 527.64 | 4502.34 | 3327.32 | -2.564102564 |
| 115.37 | 30.35 | 39.69 | 946.99 | 1310.17 | -4.166666667 |

| 304.6 | 197.35 | 262.72 | 152.31 | 232.22 | 1.87 |
| --- | --- | --- | --- | --- | --- |
| 84.01 | 232.64 | 386.84 | 619.13 | 1406.21 | 1.43 |
| 462.7 | 223.97 | 281.9 | 835.27 | 1024.7 | 1.21 |
| 1210.22 | 995.19 | 1294.26 | 547.09 | 849.06 | 1.4 |
| 168.52 | 102.08 | 130.45 | 57.1 | 82.92 | 1.32 |
| 83.55 | 350 | 269.59 | 58.09 | 118.74 | 1.94 |
| 178.28 | 126.85 | 104.71 | 118.83 | 193.45 | -1.265822785 |
| 377.47 | 169.31 | 238.67 | 254.66 | 353.3 | 1.25 |
| 131.05 | 139.91 | 116.55 | 265.12 | 171.33 | -1.219512195 |
| 103.44 | 3151.4 | 5096.9 | 78.73 | 136.14 | 1.34 |
| 17.99 | 877.54 | 1240.32 | 21 | 31.75 | 1.27 |
| 11.13 | 19.71 | 16.16 | 29.67 | 24.44 | -1.5625 |
| 406.67 | 5.56 | 9.44 | 146.07 | 361.1 | -2.564102564 |
| 26.88 | 28.03 | 21.39 | 216.15 | 317.3 | -1.694915254 |
| 113.22 | 156.8 | 189.77 | 134.67 | 69.49 | -1.470588235 |
| 37.86 | 145.42 | 83.86 | 335.11 | 190.9 | -1.538461538 |
| 167.35 | 255.53 | 184 | 248.35 | 188.46 | -2.5 |
| 165.88 | 12.67 | 9.54 | 538.98 | 344.28 | -4.761904762 |
| 14.7 | 18.07 | 22.28 | 219.78 | 359.38 | 1.21 |
| 26.27 | 352.71 | 452.71 | 170.76 | 256.33 | 1.6 |
| 48.45 | 25.08 | 32.27 | 135.95 | 229.96 | -1.470588235 |
| 74.6 | 64.91 | 46.53 | 19.1 | 23.09 | -1.428571429 |
| 123.91 | 44.28 | 31.61 | 99.04 | 74.21 | -1.886792453 |
| 14.31 | 344.39 | 589.89 | 9.33 | 14.25 | -3.448275862 |
| 606.22 | 624.2 | 754.73 | 510.73 | 673.83 | 1.32 |
| 76.38 | 5.61 | 7.74 | 69.43 | 148.77 | -1.369863014 |
| 157.36 | 11.53 | 18.87 | 225.58 | 327.66 | -4.166666667 |
| 379.19 | 105.3 | 136.32 | 475.86 | 627.18 | 1.6 |
| 47.36 | 60.25 | 40.09 | 66.35 | 82.33 | 3.01 |
| 576.48 | 119.18 | 97.46 | 601.1 | 872.34 | -2.272727273 |
| 64.23 | 49.03 | 81.76 | 46.62 | 88.24 | -2.040816327 |
| 57.44 | 62.95 | 52.06 | 112.52 | 151.8 | 1.64 |
| 920.72 | 169.01 | 277.68 | 609.19 | 823.19 | 1.26 |
| 671.6 | 364.52 | 607.38 | 475.93 | 758.37 | 1.78 |
| 973.99 | 561.36 | 730.43 | 641.77 | 926.36 | 1.65 |
| 688.31 | 403.51 | 630.28 | 435.43 | 587.15 | 1.24 |
| 448.78 | 450.46 | 555.68 | 279.71 | 482.07 | 1.53 |
| 151.95 | 154.72 | 127.28 | 188.38 | 317.32 | -1.219512195 |
| 1055.38 | 10.78 | 14.43 | 1770.89 | 1095.31 | -3.571428571 |
| 1106.56 | 9.21 | 11.22 | 1933.9 | 1100 | -3.571428571 |

| 932.06 | 89.38 | 57.61 | 5007.27 | 9782.98 | -7.142857143 |
| --- | --- | --- | --- | --- | --- |
| 151.14 | 284.34 | 203.19 | 42.31 | 33.99 | -1.234567901 |
| 113.69 | 73.78 | 90.68 | 104.14 | 180.77 | -1.219512195 |
| 164.2 | 61.74 | 74.74 | 176.51 | 217.28 | -1.204819277 |
| 87.99 | 60.42 | 111.08 | 68.92 | 106.87 | 1.67 |
| 103.07 | 917.58 | 1258.72 | 113.58 | 86.92 | 1.5 |
| 110.92 | 69.66 | 86.31 | 141.18 | 252 | 1.22 |
| 47.65 | 405.13 | 302.17 | 130.87 | 90.73 | 1.24 |
| 574.57 | 216.38 | 264.38 | 282.93 | 540.52 | 1.26 |
| 1735.87 | 1983.31 | 3089.53 | 1630.87 | 2469.22 | 1.3 |
| 647.6 | 307.4 | 397.63 | 266.28 | 198.24 | 1.33 |
| 668.62 | 90.38 | 109.19 | 462.88 | 818.25 | -1.612903226 |
| 982.01 | 32.99 | 77.17 | 137.22 | 467.38 | 1.25 |
| 107.89 | 192.07 | 157.43 | 29.74 | 36.63 | 1.22 |
| 63.78 | 107.6 | 86.66 | 84.27 | 69.88 | 1.33 |
| 491.98 | 110.64 | 66.57 | 1560.32 | 1184.5 | -2.857142857 |
| 168.21 | 152.98 | 214.47 | 123.79 | 204.88 | -1.388888889 |
| 282.51 | 162.32 | 132.54 | 2085.44 | 1578.31 | -1.886792453 |
| 342.46 | 1031.81 | 772.2 | 414.32 | 653.04 | -1.428571429 |
| 92.6 | 37.37 | 48.47 | 82.9 | 65.04 | -1.219512195 |
| 316.23 | 19.97 | 55.09 | 571.06 | 705.21 | -1.470588235 |
| 640.61 | 151.65 | 182.21 | 381.73 | 701.23 | 1.48 |
| 621.79 | 234.34 | 175.02 | 1189.51 | 2803.34 | -1.351351351 |
| 83.92 | 13.92 | 20.92 | 54.61 | 33.13 | -1.587301587 |
| 348.16 | 708.27 | 998.15 | 1523.54 | 1863.84 | 1.27 |
| 561.43 | 37.84 | 29.77 | 249.78 | 332.35 | -1.724137931 |
| 1692.33 | 859.95 | 1087.72 | 1234.05 | 1539.78 | 1.29 |
| 86.93 | 182.29 | 223.4 | 51.73 | 85.17 | 1.79 |
| 243.5 | 230.94 | 305.5 | 426.36 | 693.83 | 1.33 |
| 701.8 | 823.93 | 1103.99 | 511.87 | 881.42 | 1.21 |
| 177.94 | 62.8 | 49.92 | 1523.15 | 794.43 | -6.666666667 |
| 1243.84 | 565.49 | 710.31 | 712.68 | 982.37 | 1.22 |
| 153.17 | 17.03 | 13.81 | 13.45 | 9.96 | -16.66666667 |
| 289.39 | 108.82 | 143.16 | 129.04 | 182.86 | -1.333333333 |
| 817.41 | 391.35 | 489.25 | 961.76 | 1365.68 | 1.24 |
| 60.87 | 60.73 | 73.02 | 85.69 | 151.31 | -1.25 |
| 179.39 | 123.26 | 153.78 | 134.28 | 193.66 | 1.23 |
| 387.15 | 15.01 | 32.22 | 1512.41 | 2401.7 | -6.25 |
| 43.5 | 56.85 | 34.85 | 220.8 | 158.8 | -1.754385965 |
| 211.87 | 222.83 | 288.48 | 324.09 | 527.65 | 1.57 |

| 9.84 | 4028.55 | 5114.35 | 7.81 | 10.62 | 1.43 |
| --- | --- | --- | --- | --- | --- |
| 204.43 | 12.35 | 9.87 | 678.02 | 895.71 | -1.785714286 |
| 80.07 | 34.87 | 44.18 | 143.59 | 172.73 | -1.666666667 |
| 268.48 | 458.87 | 610.5 | 341.89 | 460.41 | 1.57 |
| 919.49 | 373.3 | 542.71 | 578.34 | 1254.34 | -1.724137931 |
| 337.1 | 14.4 | 11.46 | 645.42 | 1561.63 | -8.333333333 |
| 75.18 | 64.78 | 45.88 | 122.89 | 173.58 | -1.333333333 |
| 730.68 | 396 | 482.22 | 329.05 | 583.68 | 1.22 |
| 10.27 | 7.99 | 12.52 | 63.87 | 102.49 | 1.32 |
| 480.76 | 293.61 | 354.71 | 519.05 | 1129.77 | 3.28 |
| 63.23 | 22.85 | 31.47 | 86.17 | 52.58 | -25 |
| 760.02 | 486.33 | 750.48 | 1003.35 | 1411.76 | 1.29 |
| 141.28 | 118.55 | 87.32 | 157.36 | 205.58 | 1.27 |
| 1560.26 | 1441.19 | 1747.63 | 1178.9 | 1455.79 | 1.21 |
| 15.61 | 17.85 | 12.07 | 69.04 | 48.52 | -6.25 |
| 205.03 | 71.9 | 111.28 | 125.9 | 190.77 | 1.77 |
| 30.42 | 26.69 | 53.3 | 334.21 | 263.26 | -2.857142857 |
| 141.28 | 149.76 | 231.44 | 1001.77 | 765.59 | -1.612903226 |
| 672.39 | 763.8 | 1050.52 | 206.43 | 320.69 | 1.62 |
| 1011.63 | 1126.58 | 1964.38 | 1179.22 | 2304.28 | 2 |
| 279.5 | 204.54 | 257.42 | 691.39 | 1362.24 | -1.408450704 |
| 79.43 | 77.08 | 103.29 | 56.84 | 90.6 | 1.46 |
| 201.68 | 174.47 | 219 | 170.27 | 211.61 | 1.45 |
| 10.05 | 9.95 | 12.2 | 402.88 | 183.48 | -5.555555556 |
| 535.22 | 656.59 | 797 | 364.34 | 527.03 | 1.33 |
| 343.38 | 149.34 | 217.98 | 382.59 | 827.13 | 1.77 |
| 23.19 | 177.77 | 83.23 | 12.45 | 20.78 | -1.5625 |
| 110.28 | 28.69 | 52.91 | 156.75 | 110.31 | -6.25 |
| 365.12 | 110.22 | 89.94 | 463.31 | 198.07 | -1.666666667 |
| 57.43 | 65.4 | 80.41 | 21.99 | 49.96 | -1.315789474 |
| 115.42 | 132.12 | 177.27 | 244.58 | 383.18 | 1.37 |
| 414.4 | 91.91 | 116.32 | 752.51 | 923.4 | -1.612903226 |
| 527.48 | 187.82 | 150.24 | 276.92 | 215.42 | -2.702702703 |
| 136.1 | 49.85 | 77.46 | 50.76 | 80.61 | -1.282051282 |
| 76.08 | 597.68 | 739.9 | 15.28 | 12.71 | 1.33 |
| 57.55 | 33.8 | 47.39 | 324.54 | 472.28 | -1.612903226 |
| 133.59 | 14.12 | 19.21 | 88.66 | 118.13 | -1.449275362 |

| 871.72 | 317.92 | 468.73 | 1068.99 | 1606.92 | 1.26 |
| --- | --- | --- | --- | --- | --- |
| 88.48 | 145.08 | 114.64 | 47.67 | 36.22 | -1.492537313 |
| 768.52 | 111.32 | 83.12 | 1169.49 | 486.04 | -16.66666667 |
| 72.37 | 5.9 | 4.8 | 362.38 | 485.26 | -20 |
| 7.79 | 10.76 | 13.17 | 200.63 | 326.71 | 1.7 |
| 277.77 | 226.32 | 330.52 | 45.71 | 66.13 | 1.5 |
| 41.48 | 138.95 | 110.65 | 388.43 | 573.56 | -1.470588235 |
| 43.47 | 71.95 | 47.94 | 21.21 | 44.5 | -1.612903226 |
| 34.14 | 16.7 | 13.71 | 39.65 | 48.66 | -1.265822785 |
| 42.41 | 84.75 | 61.88 | 37.63 | 27.5 | -1.282051282 |
| 500.73 | 218.88 | 330.43 | 386.13 | 859.68 | 1.66 |
| 22.3 | 24.16 | 33.85 | 166.34 | 135.91 | -1.587301587 |
| 31.91 | 63.48 | 49 | 35.83 | 29.32 | -1.333333333 |
| 6.75 | 403.01 | 229.44 | 7.14 | 5.6 | -1.265822785 |
| 580.45 | 545.16 | 707.52 | 273.72 | 416.46 | 1.43 |
| 133.53 | 155.54 | 129.48 | 68.79 | 94.62 | -1.204819277 |
| 284.96 | 253.38 | 195.58 | 66.73 | 82.47 | 1.48 |
| 122.75 | 98.34 | 142.11 | 78.16 | 110.68 | -1.298701299 |
| 42.86 | 174.03 | 135.1 | 50.86 | 41.75 | 1.22 |
| 29.21 | 42.01 | 53.11 | 101.19 | 162.98 | 1.27 |
| 92.39 | 87.98 | 106.63 | 100.83 | 134.46 | 1.51 |
| 602.63 | 687.05 | 848.99 | 202.69 | 328.58 | 1.21 |
| 89.65 | 59.3 | 42.21 | 81.29 | 64.16 | 1.23 |
| 44.99 | 22.97 | 14.89 | 101.28 | 76.42 | -3.571428571 |
| 143.55 | 21.64 | 14.82 | 27.94 | 44.48 | -1.538461538 |
| 54.88 | 51.14 | 86.26 | 23.03 | 41.77 | 1.97 |
| 28.71 | 100.85 | 71.99 | 54.07 | 44.01 | -1.204819277 |
| 52.56 | 79.82 | 98.15 | 41.21 | 58.23 | -1.219512195 |
| 45 | 84.18 | 67.16 | 87.98 | 69.31 | -1.408450704 |
| 115.35 | 15.26 | 8.17 | 1422.77 | 1094.86 | -14.28571429 |
| 301.79 | 85.33 | 63.32 | 177.28 | 218.65 | -3.125 |
| 77.66 | 93.28 | 72.15 | 56.5 | 76.21 | -1.886792453 |
| 143.13 | 215.88 | 268.01 | 86.5 | 133.05 | 1.53 |
| 136.47 | 265.28 | 208.66 | 73.09 | 90.73 | 2.55 |
| 27.39 | 5.55 | 8.65 | 92.36 | 152.57 | -3.03030303 |
| 29.78 | 40.54 | 20.25 | 140.51 | 112.68 | -1.428571429 |
| 17.99 | 13.05 | 8.27 | 65.77 | 107.96 | -1.818181818 |

| **FC_NS2** | **FC_NS3** | **FC_NS4** | **FC_NS5** | **Symbol** | **GenBank** |
| --- | --- | --- | --- | --- | --- |

-1.282051282 1.24 -1.219512195 1.34 PXK BG573647

| -2.564102564 | 1.56 | 1.31 | 1.85 KCNE4 | NM_080671 |
| --- | --- | --- | --- | --- |
| 2.73 | -1.265822785 | -1.315789474 | -2.325581395 HS6ST2 | NM_147174 |
| -1.428571429 | -1.408450704 | -1.204819277 | -1.234567901 C1orf43 | BC008306 |
| -8.333333333 | 1.61 | 1.5 | 1.52 POSTN | AY140646 |
| 2.32 | -1.886792453 | -1.515151515 | -1.25 HHIP | AK098525 |
| 1.71 | 1.42 | 1.43 | 1.34 BICD1 | CA777994 |
| 2.09 | 1.27 | -1.282051282 | 1.42 SRGAP2 | AI263819 |
| 5.62 | 1.3 | -1.204819277 | 1.71 UG0898H09 | R41806 |
| -5.882352941 | 1.33 | 1.21 | 1.54 C1orf85 | AW090182 |
| 1.53 | 1.28 | 1.37 | 1.28 ZNF677 | AK026366 |
| 4.64 | -1.538461538 | 1.32 | -1.234567901 FLJ33534 | AK090853 |
| 2.9 | 1.5 | 1.28 | 1.21 P4HB | AK075503 |
| 1.73 | -1.492537313 | -1.428571429 | -1.851851852 ITGB5 | AL048423 |
| 2.18 | -1.282051282 | 1.32 | 1.24 ATP1B1 | NM_001677 |
| 2.98 -1.25 1.21 -1.315789474 EMP1 NM_001423 | | | | |
| -1.5625 | -1.234567901 | -1.25 | 1.21 RHEB | AW138374 |
| -1.639344262 | 1.28 | 1.37 | 1.29 SQSTM1 | NM_003900 |
| 2.11 | 1.61 | 1.85 | 1.84 FSCN1 | NM_003088 |
| 2.03 | -1.204819277 | -1.298701299 | -1.923076923 EGR1 | NM_001964 |
| -1.282051282 | 2.1 | 1.87 | 1.88 EFEMP1 | AI826799 |
| -14.28571429 | 1.52 | 1.39 | 2 PLK2 | NM_006622 |
| 2.31 1.27 -1.219512195 -1.204819277 EGFR AW157070 | | | | |
| 2.41 | 1.7 | 1.33 | 1.38 NEDD9 | AL136139 |
| -1.204819277 | 1.4 | 1.23 | 1.23 FBXO28 | NM_015176 |
| 1.39 | 1.32 | 1.43 | 1.53 UNG | NM_003362 |
| 4.39 | 1.21 | -1.492537313 | 2.12 SERPINE1 | NM_000602 |
| -2.702702703 | 1.53 | 1.6 | 1.61 ATF3 | NM_001674 |
| -1.5625 | 1.69 | 1.3 | -1.219512195 PCK2 | NM_004563 |
| -1.428571429 | 1.55 | 1.52 | 1.76 SIRPA | AB023430 |
| -1.960784314 | 1.36 | 1.21 | 1.6 RAPGEF2 | BF439282 |
| -1.694915254 | 1.34 | 1.3 | 2.49 SLC11A2 | AU154469 |
| -1.612903226 | 1.41 | 1.42 | 1.98 SLC11A2 | NM_000617 |
| 3.33 | -1.282051282 | -1.234567901 | -1.428571429 BAMBI | NM_012342 |
| 1.35 | 1.55 | 1.57 | 1.44 AP1G1 | NM_001128 |
| -1.470588235 | -1.234567901 | 1.23 | 1.24 SATB1 | NM_002971 |
| -2.380952381 | 1.43 | 1.39 | 1.32 NELL2 | NM_006159 |
| 1.78 -1.282051282 -1.388888889 -1.351351351 RTN1 NM_021136 | | | | |
| 6.4 | 1.9 | 1.31 | 1.38 ABCA1 | AF285167 |
| 1.8 | 1.23 | 1.46 | 1.79 GFAP | NM_002055 |

| -1.234567901 | -1.639344262 | -1.470588235 | -1.724137931 FCGR2A | NM_021642 |
| --- | --- | --- | --- | --- |
| 1.53 | 1.5 | 1.65 | 1.64 MID1 | NM_000381 |
| -2.325581395 | 1.82 | 1.33 | 1.52 HMOX1 | NM_002133 |
| -3.571428571 | 1.39 | 1.66 | 2.27 TPD52L1 | NM_003287 |
| 2.8 | 1.37 | 1.26 | 1.23 USP46 | BE856374 |
| -1.470588235 | 1.51 | 1.3 | 1.55 GCLM | NM_002061 |
| 1.25 | 1.25 | 1.28 | 1.45 CEP104 | NM_014704 |
| 1.34 | 1.21 | -1.298701299 | 2.04 FILIP1L | NM_014890 |
| 1.57 | 1.36 | -1.204819277 | 1.63 EML2 | NM_012155 |
| 1.46 | 1.22 | 1.41 | 1.39 NUPL1 | NM_014778 |
| -1.694915254 | -1.25 | -1.204819277 | -1.538461538 TBC1D8 | NM_007063 |
| -33.33333333 | 1.24 | 1.62 | 1.73 STC1 | AI300520 |
| -33.33333333 | 1.26 | 1.41 | 1.51 STC1 | NM_003155 |
| 2.76 | -1.785714286 | -1.219512195 | -1.219512195 ITGB3 | M35999 |
| 1.6 | 1.47 | 1.7 | 2.47 DCX | AF040254 |
| 1.28 | 1.2 | -1.315789474 | 1.47 TNFRSF11B | BF433902 |
| -1.851851852 | -1.282051282 | 1.21 | -1.923076923 NPM3 | NM_006993 |
| -2.272727273 | -1.538461538 | -1.724137931 | -1.754385965 NCAN | NM_004386 |
| 1.26 | 1.21 | -1.388888889 | -1.315789474 EGR2 | NM_000399 |
| 1.42 -1.470588235 -1.333333333 -1.5625 BBOX1 NM_003986 | | | | |
| -2.702702703 | 1.21 | 1.23 | 1.64 PLAG1 | NM_002655 |
| -4 | -1.298701299 | 1.28 | 1.5 PLAU | NM_002658 |
| 1.99 | -1.333333333 | 1.29 | 1.69 PRRX1 | NM_006902 |
| 1.3 | -1.219512195 | -1.388888889 | 1.21 EPHA3 | NM_005233 |
| -1.265822785 | -1.694915254 | -1.408450704 | -1.333333333 EGR3 | NM_004430 |
| 1.93 | 1.24 | 1.71 | 1.53 IL13RA2 | NM_000640 |
| -1.25 | 1.24 | 1.21 | 1.32 ZNF43 | NM_003423 |
| 4.12 | 1.36 | 1.38 | 2.14 PLAGL1 | NM_002656 |
| 2.38 | 1.4 | 1.64 | 1.45 MMP16 | U79292 |
| 1.86 | 1.25 | 1.29 | 1.32 EYA4 | NM_004100 |
| 1.37 | -1.25 | -1.492537313 | 1.24 SLC4A7 | NM_003615 |
| 2.31 | -1.538461538 | -1.219512195 | 1.45 STAG2 | NM_006603 |
| 1.57 | -1.470588235 | 1.67 | 1.89 AKAP13 | NM_006738 |
| -2.272727273 | 1.3 | -1.204819277 | 1.35 NR2F2 | AV703465 |
| -8.333333333 | 1.68 | 1.64 | 1.35 CRYAB | AF007162 |
| 2.23 | 1.57 | 1.67 | 1.59 SWAP70 | AI139569 |
| 1.94 | 1.27 | 1.3 | 1.44 SWAP70 | AB014540 |
| -3.03030303 | 1.53 | 1.56 | 1.35 DDIT3 | BC003637 |
| -3.03030303 | 1.41 | 1.23 | 1.72 RIN2 | AL136924 |
| 1.38 | 1.35 | -1.219512195 | 1.68 NR1D2 | N32859 |
| 7.06 | -1.428571429 | 1.34 | -1.612903226 LRRN3 | AI221950 |
| 7.63 | -1.492537313 | 1.22 | -1.754385965 LRRN3 | AL442092 |
| -3.225806452 | 1.53 | 1.51 | 2.04 SLC7A11 | AB040875 |
| -5.555555556 | -1.265822785 | -1.204819277 | 1.72 FAP | U76833 |

| -2.5 | 1.28 | 1.51 | 1.75 MALT1 | AB026118 |
| --- | --- | --- | --- | --- |
| 1.21 | -1.298701299 | -1.265822785 | -1.754385965 ARC | AF193421 |
| 2.38 | 1.45 | -1.5625 | 1.95 IGFBP3 | M31159 |
| -25 | -1.470588235 | -1.408450704 | -1.25 RGS20 | AF074979 |
| 1.28 | 1.23 | 1.23 | 1.74 PPM1A | AF070670 |
| 1.74 | 1.26 | 1.21 | 1.23 TRO | U04811 |
| 2 | 1.43 | 1.84 | 1.55 FSCN1 | BC004908 |
| -3.03030303 1.39 1.37 -1.298701299 PTGDS BC005939 | | | | |
| 2.06 | -1.388888889 | 1.24 | 1.79 C1R | AL573058 |
| -5.555555556 | 1.29 | -1.333333333 | -1.449275362 MYO1B | BF215996 |
| -1.639344262 | 1.31 | 1.22 | 1.91 ARID5B | BG285011 |
| 1.81 1.21 1.56 1.51 SERINC5 AI700633 | | | | |
| -1.351351351 | -1.25 | 1.29 | -1.351351351 COBL | AA594937 |
| 1.67 | 1.28 | 1.21 | 1.77 ST3GAL6 | AI989567 |
| 2.47 | 2.63 | 2.34 | 3.41 CNR1 | U73304 |
| -4.347826087 | 1.3 | -1.219512195 | 1.23 NFASC | AA995925 |
| 2.43 -1.219512195 -1.234567901 -1.204819277 D4S234E M98528 | | | | |
| -2.43902439 | 1.46 | -1.666666667 | -1.315789474 SLIT1 | AB011537 |
| -1.298701299 | -1.724137931 | 1.4 | 1.66 HIPK2 | R37104 |
| -2.564102564 | 2.38 | -1.219512195 | -1.315789474 TRDV2 | AW007751 |
| -1.234567901 | -1.587301587 | -1.333333333 | 1.58 C6orf62 | BE465032 |
| 2.9 | -1.449275362 | 1.3 | -1.282051282 FBXL2 | AF176518 |
| 2.01 | 1.22 | 2.76 | 1.23 CNIH3 | AF070524 |
| 3.94 | 2.36 | 1.2 | 1.84 DOK5 | AL050069 |
| 3.4 1.27 -1.333333333 2.36 ADAMTS3 AB002364 | | | | |
| 2.45 | -1.265822785 | 1.5 | -1.639344262 NTRK3 | S76476 |
| -1.298701299 | 1.75 | 1.41 | 1.22 CD24 | AK000168 |
| 1.64 | -1.265822785 | -1.265822785 | 1.33 PDGFA | X03795 |
| -3.03030303 | 1.37 | 1.26 | 1.25 HERPUD1 | AF217990 |
| -1.408450704 | 1.23 | 1.23 | 1.65 FAM55C | AA721025 |
| -3.125 | 2.07 | 1.32 | 1.63 SLC7A11 | AA488687 |
| -5 | 1.39 | 1.34 | 1.72 NAMPT | NM_005746 |
| 1.46 | -1.724137931 | -1.265822785 | -1.923076923 A2M | NM_000014 |
| -1.25 | 1.39 | 1.26 | 1.38 EPS15 | NM_001981 |
| 2.26 | 1.2 | -1.234567901 | -1.351351351 DSG2 | BF031829 |
| 1.27 | -1.282051282 | 1.32 | 1.42 ZNF22 | NM_006963 |
| 2.43 | 1.29 | 1.25 | 1.42 TMEM9B | NM_020644 |
| 1.46 | -1.298701299 | 1.2 | 1.77 EXOC5 | NM_006544 |
| -1.219512195 | 1.38 | 1.25 | 1.44 TTC17 | NM_018259 |
| 2.55 | 1.32 | 2.15 | 1.59 CSGALNACT1 | NM_018371 |
| 3.83 -1.282051282 -1.639344262 -1.388888889 OSBPL10 NM_017784 | | | | |
| -2.222222222 | 1.25 | 1.29 | 1.63 SLCO3A1 | NM_013272 |
| -12.5 | 1.22 | 1.44 | 1.29 CA14 | NM_012113 |
| 1.33 | -1.234567901 | -1.219512195 | 1.21 AGPAT4 | NM_020133 |

1.61 -1.515151515 1.23 -1.428571429 LIN7B NM_022165

| 1.47 | 1.2 | -1.298701299 | 1.39 TMC7 | NM_024847 |
| --- | --- | --- | --- | --- |
| -1.369863014 | -1.388888889 | -1.219512195 | 1.37 EDEM3 | NM_017992 |
| 3.8 | -2.083333333 | 2.01 | 1.44 C21orf62 | NM_019596 |
| 2.17 | -1.351351351 | 1.35 | -1.492537313 LEF1 | AF288571 |
| -100 | 1.55 | 1.27 | 1.36 NEFL | AL537457 |
| 2.37 | -1.587301587 | -1.25 | 1.32 AMIGO2 | AC004010 |
| 1.57 | -1.298701299 | 1.27 | 1.2 CADM4 | AW204383 |
| 1.26 | 1.25 | 1.33 | 1.35 CCDC25 | AU160632 |
| 1.54 -1.369863014 1.45 2.17 TMEM106B AV705186 | | | | |
| 2.07 | 1.53 | -1.25 | 2.42 SLC40A1 | AL136944 |
| 2.87 | -1.25 | -1.408450704 | 1.41 C21orf62 | BC004959 |
| 2.35 | 1.37 | 1.22 | 1.77 SECISBP2 | BC001189 |
| 1.24 | 1.21 | 1.57 | 1.6 AGPAT9 | BC006236 |
| -1.282051282 | 1.47 | 1.21 | 2.18 MALAT1 | AF001540 |
| -1.818181818 | -1.851851852 | 1.38 | -1.639344262 H19 | BF569051 |
| 2.1 | 1.45 | 1.54 | 1.41 NDFIP2 | AA019338 |
| 1.24 | 1.26 | -1.351351351 | 1.31 BTBD7 | AI580162 |
| 1.34 | 1.21 | 1.21 | 1.23 NRAS | BE964484 |
| -1.639344262 | -1.694915254 | -1.470588235 | -1.428571429 H19 | AL575306 |
| 2.81 | 1.46 | 1.55 | 1.52 RTKN | BC004558 |
| 1.78 | 1.63 | 2 | -1.265822785 MYO5B | AB032945 |
| 1.28 | 1.61 | 1.55 | -1.315789474 MYO5B | AI991160 |
| -2 | 1.39 | 1.38 | 1.55 FAM45A | AI697488 |
| 2.02 | 1.73 | 1.74 | 1.95 FAM108C1 | AI339710 |
| 1.35 | 1.31 | 1.26 | 1.97 NRP2 | AI819729 |
| -1.234567901 | 1.53 | 1.34 | 1.59 C8orf83 | AW303300 |
| -1.515151515 | 1.41 | 1.26 | 1.24 C8orf83 | BE962119 |
| -1.408450704 | -1.25 | 1.23 | -2.173913043 COL12A1 | AA788946 |
| -1.428571429 | 1.3 | 1.21 | 1.45 XRN1 | BG534738 |
| 2.94 | 1.56 | 1.46 | 2.16 TMOD2 | AW207699 |
| -11.11111111 | -1.666666667 | -2.127659574 | 1.67 VAT1L | AA156723 |
| 2.23 | 1.26 | 1.84 | -1.428571429 CD109 | AL110152 |
| 2.49 | -1.428571429 | -1.219512195 | -2.325581395 PLEKHH2 | AI913749 |
| -1.5625 | -1.315789474 | 1.23 | 2.27 C12orf35 | AI979334 |
| -1.587301587 | 1.42 | 1.34 | 1.57 SLCO3A1 | AW976431 |
| 1.8 | 1.3 | 1.27 | 1.23 SETBP1 | BF739885 |
| 2.06 | -1.333333333 | -1.25 | -1.282051282 SLC44A1 | AV721564 |
| 3.87 | 1.54 | 1.55 | 1.59 SGK3 | AV690866 |
| -20 | 1.84 | 1.24 | -1.204819277 IGFBPL1 | AL522781 |
| 1.47 | 1.55 | 1.4 | 1.46 FOSL2 | AI860150 |
| 2.56 | 1.52 | 1.36 | 1.33 EPB41L4A | AU144565 |
| -1.5625 | -1.886792453 | -1.612903226 | 1.55 ELAVL2 | AL161628 |
| -2.564102564 | 1.22 | 1.22 | 1.25 CDRT4 | AV702789 |

| 2.63 | -1.5625 | 1.27 | -1.369863014 PCDH10 | AI640307 |
| --- | --- | --- | --- | --- |
| 1.52 | -1.369863014 | -2.127659574 | -1.818181818 NTRK3 | AI140305 |
| 4.7 | 1.55 | 1.23 | 2.23 ADAMTS15 | AI970797 |
| 1.31 | 1.42 | 1.68 | 2.86 NRP2 | N90777 |
| -1.234567901 | -1.298701299 | 1.31 | 1.39 FNBP4 | AI083506 |
| 1.86 1.35 1.47 1.5 PROX1 BF223556 | | | | |
| -2.083333333 | 1.29 | -1.265822785 | -1.315789474 C10orf47 | AI640157 |
| 3.11 | -1.25 | -1.333333333 | -2.380952381 HS6ST2 | AI767756 |
| 1.97 | -1.428571429 | -1.234567901 | 1.34 GRIA3 | AW294729 |
| -1.492537313 | -1.666666667 | 1.22 | 1.63 F2RL2 | AI378647 |
| 3.47 | 1.4 | 1.46 | 1.45 EPHB1 | AI674183 |
| 1.94 | 1.2 | -1.25 | 1.48 KCNJ2 | BF111326 |
| -1.470588235 | -1.724137931 | -1.492537313 | 2.1 ETNK1 | AA829940 |
| 1.24 | 1.22 | -1.219512195 | 1.23 VMP1 | AV660825 |
| 3.68 -1.298701299 -1.369863014 -1.369863014 CAPS NM_004058 | | | | |
| 1.31 | 1.51 | 1.51 | 2.23 MALAT1 | NM_014086 |
| -5.882352941 | 1.46 | 1.4 | -1.219512195 RUNX2 | AL353944 |
| 5.19 | -1.298701299 | -1.298701299 | -1.219512195 C9orf3 | AL137535 |
| -20 | -1.25 | -1.754385965 | -1.282051282 RALGAPA2 | AB033098 |
| -1.851851852 | 1.38 | 1.3 | 1.52 GCLM | AA630626 |
| -1.369863014 | -1.315789474 | -1.204819277 | 1.38 ADCY1 | AA021120 |
| -2.127659574 | 1.55 | -1.298701299 | 1.24 ITPKB | AA348410 |
| -1.587301587 | -1.204819277 | 1.45 | 1.42 FKBP14 | BG110260 |
| -2.777777778 | -1.265822785 | -1.282051282 | -1.219512195 WDR4 | AA555280 |
| -2.083333333 | -1.470588235 | 1.26 | 1.61 XKR6 | AA742584 |
| -1.5625 | 1.67 | 1.21 | 1.33 ZNF254 | AI983886 |
| -1.886792453 | 1.5 | 1.24 | 1.62 GCLM | AI753488 |
| 1.64 | -1.818181818 | -1.408450704 | -1.265822785 RDH5 | AI887702 |
| 2.42 | -1.315789474 | -1.538461538 | -1.333333333 PDE1C | AW299452 |
| 1.29 | 1.49 | -1.470588235 | 1.59 SNCAIP | BF062366 |
| -1.351351351 | 1.62 | 1.69 | 1.81 IPMK | AW902062 |
| -2.777777778 | -1.666666667 | -1.408450704 | -1.234567901 LOC100505730 | BG028463 |
| 1.49 | -1.204819277 | 1.23 | 1.41 CRLS1 | AI339837 |
| 2.58 | -1.5625 | -1.25 | -1.265822785 LMO4 | R13594 |
| 3.23 | -1.785714286 | -1.851851852 | -1.298701299 CHRM3 | R55784 |
| 5.31 | 1.28 | -1.351351351 | 1.23 LPPR5 | AL526459 |
| -1.388888889 | -1.388888889 | -1.298701299 | 1.35 ZNF652 | AL036450 |
| -1.538461538 | 1.33 | 1.24 | 1.54 FAM55C | BE883167 |
| -1.538461538 | -1.428571429 | -1.265822785 | 1.24 TFDP2 | AI049624 |
| 3.7 | -1.351351351 | 1.56 | 1.65 SEMA3A | BF215018 |
| 4.73 | -1.587301587 | -2 | -1.25 SYT6 | AI863338 |
| 6 | -1.333333333 | -1.587301587 | 1.64 SEMA3A | BF102683 |

**Description**

PX domain containing serine/threonine kinase

potassium voltage-gated channel, Isk-related family, member 4 heparan sulfate 6-O-sulfotransferase 2

chromosome 1 open reading frame 43 periostin, osteoblast specific factor hedgehog interacting protein

bicaudal D homolog 1 (Drosophila)

SLIT-ROBO Rho GTPase activating protein 2 uncharacterized LOC643763

chromosome 1 open reading frame 85 zinc finger protein 677

uncharacterized LOC285150

prolyl 4-hydroxylase, beta polypeptide integrin, beta 5

ATPase, Na+/K+ transporting, beta 1 polypeptide epithelial membrane protein 1

Ras homolog enriched in brain sequestosome 1

fascin homolog 1, actin-bundling protein (Strongylocentrotus purpuratus)

early growth response 1

EGF containing fibulin-like extracellular matrix protein 1 polo-like kinase 2

epidermal growth factor receptor

neural precursor cell expressed, developmentally down-regulated 9

F-box protein 28

uracil-DNA glycosylase

serpin peptidase inhibitor, clade E (nexin, plasminogen activator inhibitor type 1), member 1 activating transcription factor 3

phosphoenolpyruvate carboxykinase 2 (mitochondrial)

signal-regulatory protein alpha

Rap guanine nucleotide exchange factor (GEF) 2

solute carrier family 11 (proton-coupled divalent metal ion transporters), member 2 solute carrier family 11 (proton-coupled divalent metal ion transporters), member 2

BMP and activin membrane-bound inhibitor homolog (Xenopus laevis)

adaptor-related protein complex 1, gamma 1 subunit

SATB homeobox 1

NEL-like 2 (chicken)

reticulon 1

ATP-binding cassette, sub-family A (ABC1), member 1 glial fibrillary acidic protein

Fc fragment of IgG, low affinity IIa, receptor (CD32)

midline 1 (Opitz/BBB syndrome) heme oxygenase (decycling) 1 tumor protein D52-like 1 ubiquitin specific peptidase 46

glutamate-cysteine ligase, modifier subunit centrosomal protein 104kDa

filamin A interacting protein 1-like

echinoderm microtubule associated protein like 2 nucleoporin like 1

TBC1 domain family, member 8 (with GRAM domain)

stanniocalcin 1 stanniocalcin 1

integrin, beta 3 (platelet glycoprotein IIIa, antigen CD61)

doublecortin

tumor necrosis factor receptor superfamily, member 11b nucleophosmin/nucleoplasmin 3

neurocan

early growth response 2

butyrobetaine (gamma), 2-oxoglutarate dioxygenase (gamma-butyrobetaine hydroxylase) 1 pleiomorphic adenoma gene 1

plasminogen activator, urokinase paired related homeobox 1

EPH receptor A3

early growth response 3 interleukin 13 receptor, alpha 2 zinc finger protein 43

pleiomorphic adenoma gene-like 1

matrix metallopeptidase 16 (membrane-inserted)

eyes absent homolog 4 (Drosophila)

solute carrier family 4, sodium bicarbonate cotransporter, member 7 stromal antigen 2

A kinase (PRKA) anchor protein 13

nuclear receptor subfamily 2, group F, member 2 crystallin, alpha B

SWAP switching B-cell complex 70kDa subunit SWAP switching B-cell complex 70kDa subunit DNA-damage-inducible transcript 3

Ras and Rab interactor 2

nuclear receptor subfamily 1, group D, member 2 leucine rich repeat neuronal 3

leucine rich repeat neuronal 3

solute carrier family 7 (anionic amino acid transporter light chain, xc- system), member 11 fibroblast activation protein, alpha

mucosa associated lymphoid tissue lymphoma translocation gene 1 activity-regulated cytoskeleton-associated protein

insulin-like growth factor binding protein 3 regulator of G-protein signaling 20

protein phosphatase, Mg2+/Mn2+ dependent, 1A

trophinin

fascin homolog 1, actin-bundling protein (Strongylocentrotus purpuratus)

prostaglandin D2 synthase 21kDa (brain) complement component 1, r subcomponent myosin IB

AT rich interactive domain 5B (MRF1-like)

serine incorporator 5

cordon-bleu homolog (mouse)

ST3 beta-galactoside alpha-2,3-sialyltransferase 6 cannabinoid receptor 1 (brain)

neurofascin

DNA segment on chromosome 4 (unique) 234 expressed sequence slit homolog 1 (Drosophila)

homeodomain interacting protein kinase 2

T cell receptor delta variable 2 chromosome 6 open reading frame 62

F-box and leucine-rich repeat protein 2 cornichon homolog 3 (Drosophila) docking protein 5

ADAM metallopeptidase with thrombospondin type 1 motif, 3 neurotrophic tyrosine kinase, receptor, type 3

CD24 molecule

platelet-derived growth factor alpha polypeptide

homocysteine-inducible, endoplasmic reticulum stress-inducible, ubiquitin-like domain member 1 family with sequence similarity 55, member C

solute carrier family 7 (anionic amino acid transporter light chain, xc- system), member 11 nicotinamide phosphoribosyltransferase

alpha-2-macroglobulin

epidermal growth factor receptor pathway substrate 15 desmoglein 2

zinc finger protein 22 (KOX 15) TMEM9 domain family, member B exocyst complex component 5 tetratricopeptide repeat domain 17

chondroitin sulfate N-acetylgalactosaminyltransferase 1 oxysterol binding protein-like 10

solute carrier organic anion transporter family, member 3A1 carbonic anhydrase XIV

1-acylglycerol-3-phosphate O-acyltransferase 4 (lysophosphatidic acid acyltransferase, delta)

lin-7 homolog B (C. elegans)

transmembrane channel-like 7

ER degradation enhancer, mannosidase alpha-like 3 chromosome 21 open reading frame 62

lymphoid enhancer-binding factor 1 neurofilament, light polypeptide adhesion molecule with Ig-like domain 2 cell adhesion molecule 4

coiled-coil domain containing 25 transmembrane protein 106B

solute carrier family 40 (iron-regulated transporter), member 1 chromosome 21 open reading frame 62

SECIS binding protein 2

1-acylglycerol-3-phosphate O-acyltransferase 9

metastasis associated lung adenocarcinoma transcript 1 (non-protein coding) H19, imprinted maternally expressed transcript (non-protein coding)

Nedd4 family interacting protein 2

BTB (POZ) domain containing 7

neuroblastoma RAS viral (v-ras) oncogene homolog

H19, imprinted maternally expressed transcript (non-protein coding)

rhotekin myosin VB myosin VB

family with sequence similarity 45, member A family with sequence similarity 108, member C1 neuropilin 2

chromosome 8 open reading frame 83 chromosome 8 open reading frame 83 collagen, type XII, alpha 1

5'-3' exoribonuclease 1 tropomodulin 2 (neuronal)

vesicle amine transport protein 1 homolog (T. californica)-like

CD109 molecule

pleckstrin homology domain containing, family H (with MyTH4 domain) member 2 chromosome 12 open reading frame 35

solute carrier organic anion transporter family, member 3A1

SET binding protein 1

solute carrier family 44, member 1

serum/glucocorticoid regulated kinase family, member 3 insulin-like growth factor binding protein-like 1

FOS-like antigen 2

erythrocyte membrane protein band 4.1 like 4A

ELAV (embryonic lethal, abnormal vision, Drosophila)-like 2 (Hu antigen B) CMT1A duplicated region transcript 4

protocadherin 10

neurotrophic tyrosine kinase, receptor, type 3

ADAM metallopeptidase with thrombospondin type 1 motif, 15 neuropilin 2

formin binding protein 4 prospero homeobox 1

chromosome 10 open reading frame 47 heparan sulfate 6-O-sulfotransferase 2 glutamate receptor, ionotropic, AMPA 3 coagulation factor II (thrombin) receptor-like 2

EPH receptor B1

potassium inwardly-rectifying channel, subfamily J, member 2 ethanolamine kinase 1

vacuole membrane protein 1 calcyphosine

metastasis associated lung adenocarcinoma transcript 1 (non-protein coding)

runt-related transcription factor 2 chromosome 9 open reading frame 3

Ral GTPase activating protein, alpha subunit 2 (catalytic)

glutamate-cysteine ligase, modifier subunit adenylate cyclase 1 (brain)

inositol-trisphosphate 3-kinase B FK506 binding protein 14, 22 kDa WD repeat domain 4

XK, Kell blood group complex subunit-related family, member 6 zinc finger protein 254

glutamate-cysteine ligase, modifier subunit retinol dehydrogenase 5 (11-cis/9-cis) phosphodiesterase 1C, calmodulin-dependent 70kDa synuclein, alpha interacting protein

inositol polyphosphate multikinase uncharacterized LOC100505730 cardiolipin synthase 1

LIM domain only 4

cholinergic receptor, muscarinic 3

lipid phosphate phosphatase-related protein type 5 zinc finger protein 652

family with sequence similarity 55, member C

transcription factor Dp-2 (E2F dimerization partner 2)

sema domain, immunoglobulin domain (Ig), short basic domain, secreted, (semaphorin) 3A

synaptotagmin VI

sema domain, immunoglobulin domain (Ig), short basic domain, secreted, (semaphorin) 3A

Supplementary Table2:

**Symbol Description GenBank Gene Ontology Biological Process**

| AGPAT9 | 1-acylglycerol-3-phosphate O-aBC006236 | intracellular signal transduction regulation of signal transduction signal transduction |
| --- | --- | --- |
| DOK5 | docking protein 5 AL050069 | intracellular signal transduction signal transduction  nervous system development |
| EPHB1 | EPH receptor B1 AI674183 | intracellular signal transduction regulation of signal transduction signal transduction  central nervous system projection neu central nervous system neuron axonog central nervous system neuron develo central nervous system neuron differe regulation of nervous system develop central nervous system development neurological system process  nervous system development |
| NDFIP2 | Nedd4 family interacting proteAA019338 | positive regulation of signal transductio intracellular signal transduction regulation of signal transduction  signal transduction |
| NEDD9 | neural precursor cell expresse AL136139 | signal transduction |
| NRAS | neuroblastoma RAS viral (v-ras BE964484 | positive regulation of Ras protein signa positive regulation of small GTPase me regulation of Ras protein signal transdu Ras protein signal transduction regulation of small GTPase mediated s small GTPase mediated signal transdu positive regulation of signal transductio intracellular signal transduction regulation of signal transduction leukocyte migration  cell migration  regulation of neurological system proc |

|  | | neurological system process nervous system development |
| --- | --- | --- |
| RALGAPA2 | Ral GTPase activating protein, AB033098 | regulation of Ras protein signal transd regulation of small GTPase mediated s small GTPase mediated signal transdu intracellular signal transduction regulation of signal transduction  signal transduction |
| RTKN | rhotekin BC004558 | Rho protein signal transduction  Ras protein signal transduction  small GTPase mediated signal transdu intracellular signal transduction  signal transduction regulation of anti-apoptosis Anti-apoptosis |
| TMEM9B | TMEM9 domain family, memb NM_020644 | positive regulation of signal transducti intracellular signal transduction regulation of signal transduction  signal transduction |
| PROX1 | prospero homeobox 1 BF223556 | positive regulation of endothelial cell regulation of endothelial cell migration cell migration  stem cell differentiation  central nervous system neuron differe regulation of nervous system develop central nervous system development nervous system development |
| FSCN1 | fascin homolog 1, actin-bundli NM_003088 | cell migration |
| EGR3 | early growth response 3 NM_004430 | neurological system process nervous system development |

**Trend P-Value Reference**

- 0.009719381

- 1.12E-005hang J, et al. 200

- 1.76E-006

- 0.009719381

- 1.76E-006

- 0.00029

Shi et al. 2006

- 0.009719381

- 1.12E-005

- 1.76E-006

- 0.02028

- 0.03111

- 0.13237 l. 2003; Nakada

- 0.00674

- 0.05817

- 0.05416

- 0.00000

- 0.00029

- 0.000625589

- 0.009719381

- 1.12E-005

- 1.76E-006

Mund 2010

- 1.76E-006 t al 2010; Kim M et al 2006

- 0.043705151

- 0.04825983

- 0.049325487

- 0.055696102

- 0.03439581

- 0.006769571

- 0.000625589 al 2004; Tsao H

- 0.009719381

- 1.12E-005

- 0.002078

- 0.000000

- 0.04826

| - | 0.00000 |
| --- | --- |
| - | 0.00029 |
| + | 0.049325487 |
| + | 0.03439581 |

+ 0.006769571 aito R et al 201

+ 0.009719381

+ 1.12E-005

+ 1.76E-006

- 0.217214981

- 0.055696102

- 0.006769571

- 0.009719381 Wang et al 2008

- 1.76E-006

- 0.145194489

- 0.114230438

- 0.000625589

- 0.009719381odeller et al. 20

- 1.12E-005

- 1.76E-006

- 0.005791

- 0.003159

- 0.000000

- 0.28174

- 0.00674

- 0.05817

- 0.05416

- 0.00029

Elsir et al 2010

- 0.000000 Chiyomaru et al 2010; Fuse et al 2011

+ 0.00000

+ 0.00029

2004; Rickman
