# Supplementary material for: NEDD9, a novel target of miR-145, increases the invasiveness of glioblastoma
Source: Oncotarget. 2012 Aug 5;3(7):723–34. doi: 10.18632/oncotarget.547 (PMC3443255; doi:10.18632/oncotarget.547)
Supplement: Supplementary file 2 [file oncotarget-03-723-s002.pdf]

NEDD9, a novel target of miR-145, increases the invasiveness of glioblastoma - Speranza et al

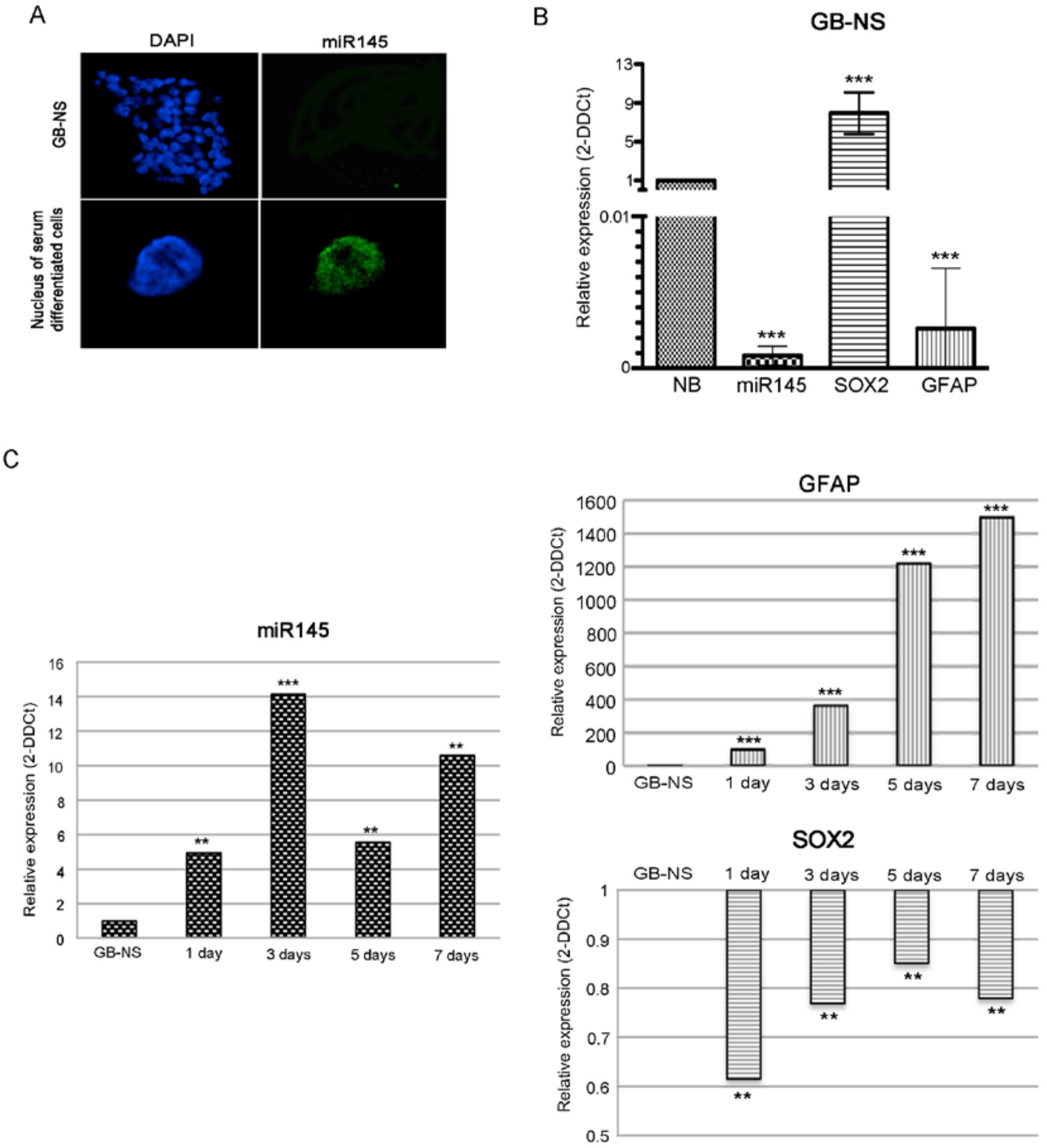

Supplementary Figure S1. A) FISH analysis confirmed the absence of miR145 expression in GB-NS as well as the increased levels of miR145 after differentiation in the presence of serum (GB-NS 6 days after 3% serum differentiation). B) Real-time PCR analysis was used to assess the correlations between the levels of miR145, SOX2 and GFAP expression in GB-NS. We observed a high level of SOX2 expression and very low levels of miR145 and GFAP expression compared to normal brain (** $P < 0.01$; *** $P < 0.001$). The histograms represent the mean ± SD of three different GB-NS cell lines compared to normal brain (miR145, mean ± SD: 0.00083 ± 0.00062; SOX2, mean ± SD: 7.94 ± 2.15, GFAP, mean ± SD: 0.0026 ± 0.0039). SOX2 and GFAP were normalized to the housekeeping gene beta-2 microglobulin, and miR145 expression was normalized to RNU6B. C) Real-time PCR analysis was performed using GB-NS cell lines before and after differentiation (1, 3, 5 and 7 days), and the results showed that miR145 and GFAP were up-regulated and that SOX2 was down-regulated. The histograms represent the mean ± SD of three different GB-NS cell lines (* $P < 0.05$; ** $P < 0.01$; *** $P < 0.001$).

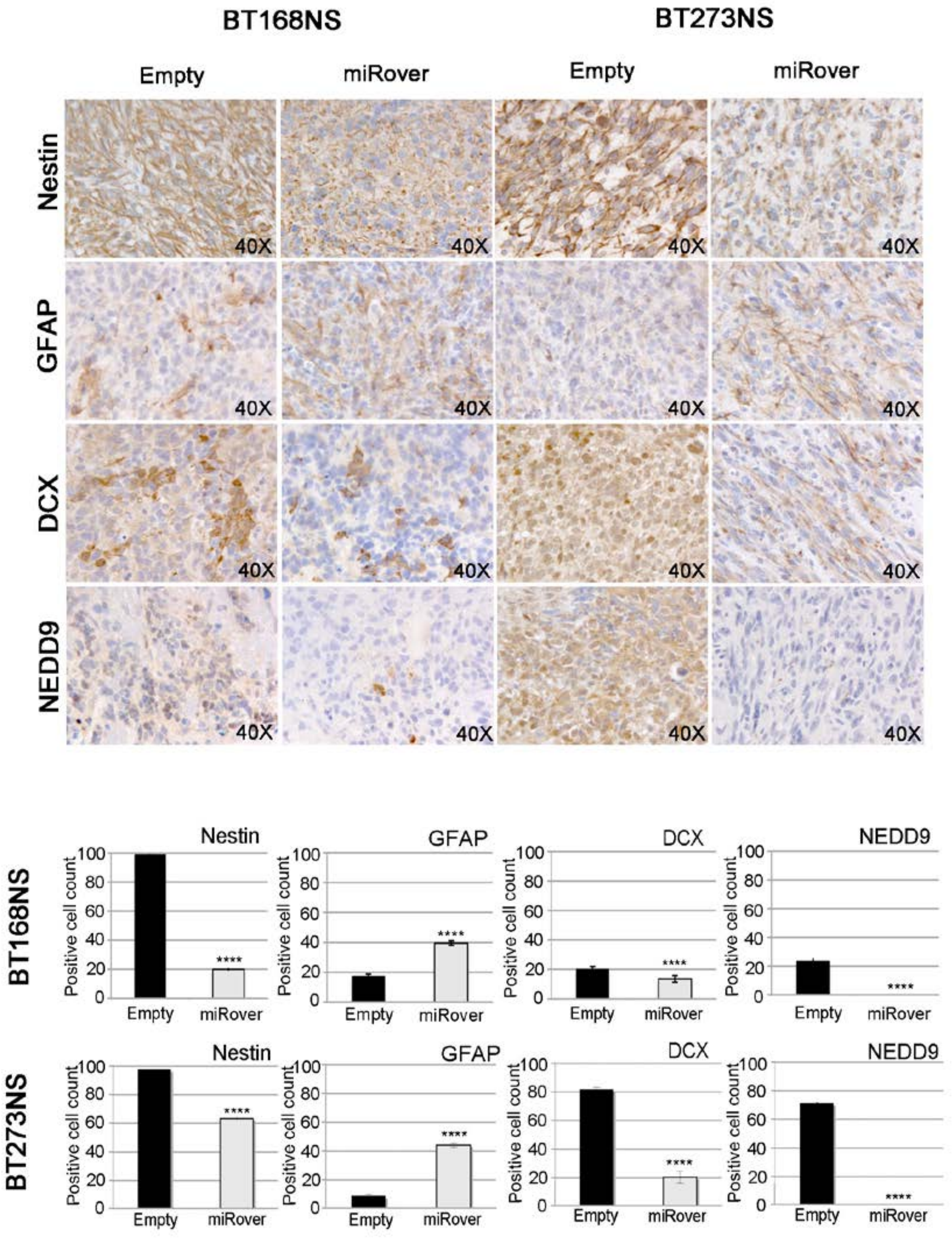

Supplementary Figure S2. Histological analysis performed on miRover and Empty tumors (40X) of two different cell lines (BT168NS and BT273NS). miRover tumors had a greater amount of GFAP-positive cells and lower amounts of cells positive for Nestin, DCX and NEDD9 compared to Empty tumors. The histograms represent the immunohistochemical quantification of Nestin, GFAP, DCX, and NEDD9 in miRover tumors compared to empty tumors. Positive cell counting was performed on five independent 40X fields. Three mice from each group were analyzed, and representative images for each type of tumor are displayed. Staining was performed on adjacent sections of paraffin-embedded brain samples (* P < 0.05; *** P < 0.001; **** P < 0.0001).

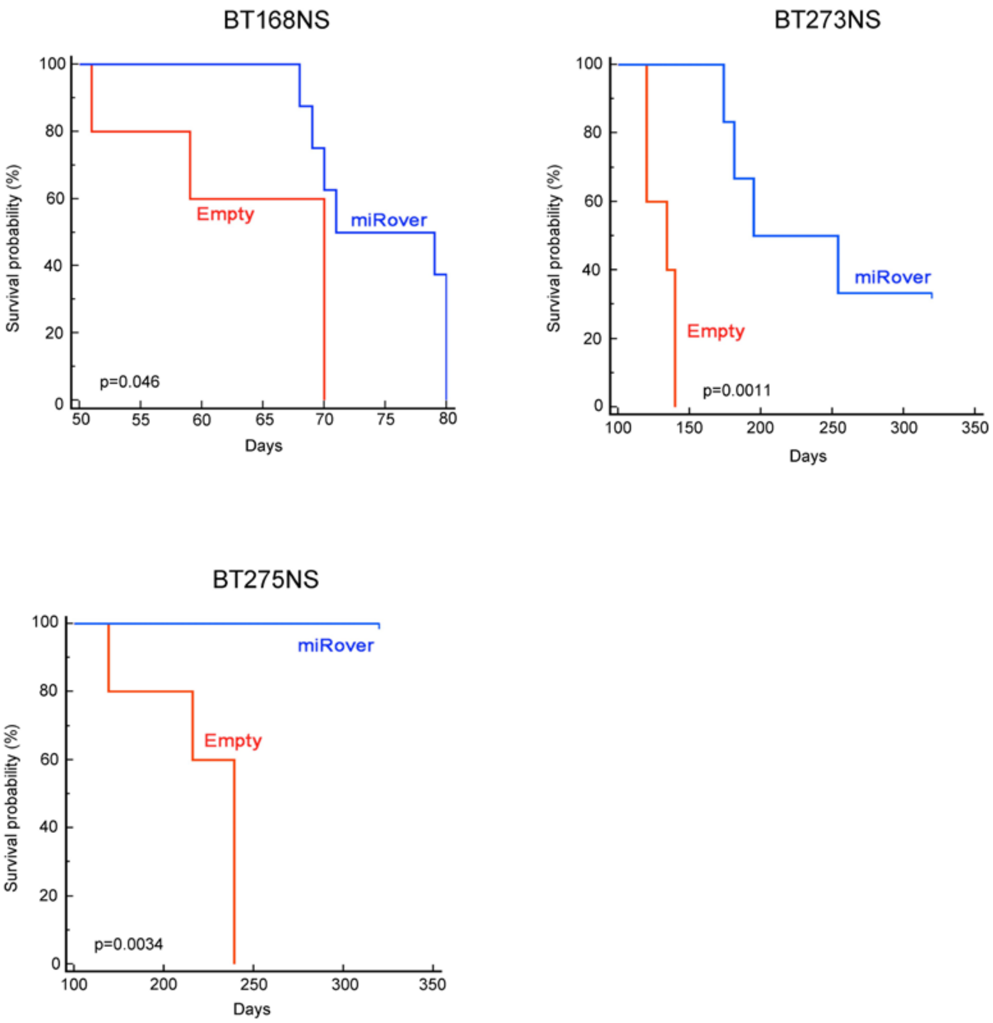

Supplementary Figure S3. Kaplan-Meier survival analysis of immunodeficient mice injected with 10^5 NS cells (BT165, BT168, BT273 and BT275) and infected with the pCLNSX-miR145 retroviral vector (n = 10 mice) or pCLNSX-Empty retroviral vector (n = 5 mice). Mice injected with miRover survived significantly longer than mice injected with Empty.

Supplementary Methods.
FISH: Non-radioactive in situ hybridization for miR145 was performed using the FITC-labeled miRCURY^{TM} LNA microRNA detection probe (Exiqon, Vedbaek, Denmark).
Deparaffinized and rehydrated tumor sections (5 microm) were treated with proteinase K (10 microg/ml; Qiagen) at 37°C for 5 min and then fixed with 4% paraformaldehyde for 10 min. All slides were placed in a Hybrite unit (Vysis) and incubated in a pre-hybridization solution at 60°C for 30 min followed by incubation in a hybridization solution with miR145 FITC-labeled probe at 60°C for 60 min. Stringent washes were performed in pre-heated 0.1X SSC buffer at 65°C, and the slides were placed in 0.2X SSC buffer at room temperature for 5 min. Tyramide signal amplification (PerkinElmer Life and Analytic Sciences Inc., Shelton, USA) was used to enhance the fluorescent signal. After quenching the endogenous peroxidase activity, slides were blocked for 30 min in blocking buffer solution and then incubated with anti FITC-HRP (1:400, DAKO) for 30 min. After washing in TNT buffer, tumor sections were incubated in a Fluorophore Tyramide Amplification Reagent working solution for 10 min. The slides were counterstained with DAPI (4′,6-diamidino-2-phenylindole, Sigma) and examined using a LEICA SP2 confocal microscope.
